# Risk factors for loneliness: A literature review

**DOI:** 10.1016/j.socscimed.2023.116163

**Published:** 2023-10

**Authors:** Martina Barjaková, Andrea Garnero, Béatrice d’Hombres

**Affiliations:** a Department of Psychology, University of Milan-Bicocca, Italy; b Directorate for Employment, Labour and Social Affairs, OECD, France; c European Commission, Joint Research Centre (JRC), Italy

**Keywords:** Individual risk factors for loneliness, Societal risk factors for loneliness, Literature review, Loneliness and COVID-19

## Abstract

**Rationale:**

Increasingly, loneliness is being recognised as a serious problem with detrimental effects on health, as well as on social cohesion and community trust. To effectively tackle this complex issue, a clear understanding of the phenomenon and its main drivers is needed. Over years of scientific research on loneliness, many potential risk factors have emerged and been tested empirically.

**Objective:**

This narrative review of 109 studies provides a concise summary of empirical evidence on the main potential risk factors for loneliness and presents an additional section dedicated to the COVID-19 pandemic.

**Method:**

Given the very large number of existing studies, emphasis is placed on recent meta-analyses and systematic literature reviews as well as longitudinal studies. Similarly, given the large number of possible risk factors for loneliness, which may differ based on the geographical and cultural context, this review focuses on studies from Europe and North America.

**Results:**

The results show that demographic factors often correlate with loneliness, but in many cases the link becomes negligible when controlling for other factors. Often, physical and mental health problems are found to be associated with loneliness, and so are some psychological factors, such as neuroticism or extroversion. Loneliness also depends on the environment in which one lives, and possibly the broader socio-economic and socio-cultural contexts. Nevertheless, the review shows that ultimately everything comes down to the quantity and quality of social relationships. In particular, marital status, living arrangements and the characteristics of one's personal social network are quite consistently found to be among the strongest predictors of loneliness. These main findings about the risk factors for loneliness remained valid also during the COVID-19 pandemic.

**Policy implications:**

The findings of this review have implications for policy, as understanding who the most vulnerable groups are is key for designing targeted policy solutions that tackle loneliness.

## Highlights

• A review of over one hundred articles examining risk factors for loneliness
• Socio-demographic factors have mostly indirect links to loneliness
• Social network characteristics and partner status matter the most for loneliness
• Many risk factors for loneliness are interrelated and impact each other
• Risk factors for loneliness remained roughly the same during the COVID-19 pandemic

## 1 Introduction

Loneliness has come to be recognised as a serious problem, and it has become even more relevant during the recent COVID-19 pandemic, as people were confined to their homes for extensive periods of time and unable to pursue their social lives as before. A recent meta-analysis of the prevalence of loneliness in 113 countries argues that *“loneliness at a problematic level is a common experience worldwide”* (Surkalim et al., 2022). For instance, the prevalence of loneliness among European adults ranged roughly between 3% and 21% before the COVID-19 pandemic, while it reached around 9–14% among adolescents in different world regions (Surkalim et al., 2022). According to another recent meta-analysis, loneliness increased significantly during the pandemic, even though the overall effect was heterogeneous and rather small - 0.27 (95% CI = [0.14–0.40]) for the standardised mean differences in continuous loneliness scores and 0.33 (95% CI = [0.04–0.62]) for the prevalence rates of loneliness (Ernst et al., 2022).

Previous studies have shown the importance of loneliness for individual wellbeing and social cohesion. Loneliness compares to obesity and smoking in its mortality risks (Baarck et al., 2021; Cuccu and Stepanova, 2021) and is associated with health problems as well as lower cognitive performance and risky behaviours (Cacioppo and Patrick, 2008).

Addressing this problem needs to be based on a clear understanding of the phenomenon and its main drivers. In particular, there needs to be awareness of the population groups that are more at risk of loneliness and the reasons for this, so that they can be targeted by specific policies. What causes loneliness also needs to be understood, in order to propose solutions that tackle the issue effectively.

Over the years of scientific research on loneliness, many potential risk factors for loneliness have been tested empirically (to varying degrees), resulting in a large body of evidence that can be difficult to navigate. Several literature reviews and meta-analyses have attempted to summarise this evidence (e.g., Cohen-Mansfield et al., 2016; Pinquart and Sörensen, 2001). They differ in their specific aims, namely in terms of the age groups and risk factors. While some of these studies summarise evidence across the whole age spectrum (e.g., Buecker et al., 2020; Morrish and Medina-Lara, 2021), others focus exclusively on older adults (e.g., Dahlberg et al., 2021; Lyu and Forsyth, 2021), or children and adolescents (e.g., Mahon et al., 2006; Schwartz-Mette et al., 2020). As for the risk factors of interest, a few studies try to capture a broad range and categorise them (e.g., Cohen-Mansfield et al., 2016; Pinquart and Sörensen, 2001), but there are perhaps more focusing on one specific factor or a narrowly defined group of factors (e.g., age (Mund et al., 2020), sex (Maes et al., 2019) or the Big Five personality traits (Buecker et al., 2020)).

To the best of our knowledge, there are no review studies that put together different categories of risk factors and focus on the whole age range of the population, which is a crucial exercise in order to understand the interrelations between different types of risk factors and identify the groups of population most at risk of loneliness. The current review fills this gap by reviewing and summarising the existing evidence on the main proposed risk factors for loneliness while also categorising them according to existing theoretical frameworks, thus producing a useful and easy-to-navigate overview of the most recent research findings on risk factors for loneliness. It also covers the whole age range of the population where evidence is available.

For this aim, more than one hundred studies were reviewed. The focus was on Europe and North America, in order to keep the review manageable but also because risk factors for loneliness may vary significantly across macro-regions that differ greatly in both socio-economic and cultural factors. The review is narrative in nature, distilling the existing knowledge in a comprehensive and critical way.

This review was initiated at a very specific time in history – during the COVID-19 pandemic, which, as mentioned above, had an impact on loneliness. It disrupted the social, family, and work lives of virtually all the people around the globe in unprecedented ways. However, this disruption affected different parts of the population in different ways, so it can be expected that the groups most vulnerable to being lonely during the pandemic would not necessarily be the same as before. Keeping in mind that a clear understanding of the risk factors is crucial to design effective policies, it is important to understand these changing dynamics. Therefore, our review includes also a section dedicated to risk factors for loneliness during the COVID-19 pandemic.

The rest of the review is structured as follows. The Methodology section explains the theoretical frameworks and the methodology used in the review. The Results section presents and discusses the findings of the review (thus, there is no separate Discussion section), splitting them into subsections about individual risk factors for loneliness, societal risk factors for loneliness, and risk factors for loneliness during the COVID-19 pandemic. Finally, the Conclusions section briefly summarises the main findings and offers some concluding remarks.

## 2 Methods

### 2.1 Definition of loneliness

In the literature, loneliness has been defined as a subjective feeling of deficiencies in one's network of social relationships (Perlman and Peplau, 1981). These deficiencies can be both quantitative and qualitative; that is, loneliness may not only arise from having too few social contacts *per se*, but also from the perception that these relationships are not satisfying enough. In other words, loneliness does not equal being alone, but feeling alone. As such, loneliness is lived as a negative experience and it is different from “solitude”, which corresponds to the voluntary and peaceful act of being alone, as well as from “social isolation”, which describes an objective state (i.e., the absence of relationships with other people and/or a very small number of meaningful ties). In the review we adopt this definition of loneliness, hence do not analyse the risk factors for social isolation or solitude.

### 2.2 Theoretical frameworks

Over many years of research on loneliness, a myriad of potential individual risk factors has been studied empirically. Obviously, not all of them impact loneliness in the same way. Hawkley et al. (2008) proposed several categories of individual risk factors for loneliness — distal, structural, and proximal — and suggested that the influence of distal and structural factors on loneliness is shaped by the more proximal factors. Specifically, the authors argue that ‘distal demographic factors (age, gender, race/ethnicity) operate through structural factors (income, education) and in turn through health, social roles, and stress — proximal factors that are more directly associated with social network size and relationship quality’ (Hawkley et al., 2008). The implied assumption is that it is these last two elements that ultimately channel the influence of distal factors on loneliness.

While useful, this framework may not capture the whole picture, as it focuses solely on individual factors. Loneliness, while being a strictly individual experience, happens within a wider societal context and may be shaped by it. For example, individual expectations of social relations are one of the ingredients for feelings of loneliness. It is easy to imagine how such expectations are formed within a specific culture, in line with its normative views about relationship standards. Therefore, the same objective individual experiences may have different impacts on loneliness, depending on local cultural norms.

Hence, a framework that incorporates such socio-economic and cultural factors with various individual factors is needed to truly understand the dynamics behind feelings of loneliness (de Jong Gierveld et al., 2006). Studying societal factors and their interaction with individual determinants of loneliness can also be useful to understand the cross-country differences in loneliness. Dykstra (2009), for instance, proposed that cross-country differences in the prevalence of loneliness among older adults may be due to (i) a different prevalence of individual factors that impact loneliness, (ii) broader differences between countries (e.g., income inequalities, societal welfare or even attitudes towards loneliness), or (iii) an interaction between the two, meaning that the importance of individual factors will vary depending on the country. de Jong Gierveld and Tesch-Römer (2012) also created a theoretical framework to help explain cross-country differences in loneliness in older adults in Europe, taking account of both individual and societal factors. They suggested that at the individual level, loneliness is determined by the quality of living conditions and the level of social integration, and that these relationships are also affected by individual social expectations. Societal factors (strength of societal welfare, demographic composition, and cultural norms and values) then shape loneliness indirectly, through individual factors.

To sum up, loneliness is thought to be influenced by both individual and societal factors. However, not all risk factors are expected to have a direct link to loneliness, but to operate through others. In this review, we broadly follow the theoretical frameworks presented above to categorise risk factors for loneliness. We provide a summary of empirical evidence on both individual and societal factors, and arrange individual factors in order from distal to more proximal ones.

### 2.3 Search and inclusion criteria

The primary search for articles was performed between September and November 2021. We first conducted a generic search using *“(“risk factors” OR determinants OR predictors OR correlates) AND loneliness”* on Google Scholar, ScienceDirect, PubMed, JSTOR, and PsycINFO, which pointed us mostly towards articles examining larger numbers of risk factors and thus allowed us to make an initial list of possible predictors. A search with more specific keywords (listed in Table A1in Supplementary Materials) was then conducted for each of these factors, to identify articles focusing on these specific topics. We complemented the online search with articles that had been used for a previous research project on risk factors of loneliness and research that was cited by others already included in our pool. The same search was then reconducted in March 2023 in order to update the review. This strategy provided us with a substantial number of articles (over 300 overall). These were then filtered through the following criteria to get the final pool of 109 articles included in the review (81 articles from the primary search and 28 articles from the second search):

1. We prioritised meta-analyses and (systematic) reviews of literature as they themselves provide a robust overview of existing evidence up to a certain point. Where available, we essentially base our discussion on these and complement it with more recent individual studies;
2. We included only quantitative studies;
3. We prioritised studies published in peer-reviewed journals;
4. We focused on more recent studies, as loneliness research spans several decades, by excluding articles published before 2000;
5. We focused on studies covering Europe and North America, as it is possible that risk factors for loneliness change based on geographical location;
6. Where possible, we prioritised studies that focus on the specific risk factor, rather than using it merely as a control variable;
7. We did not discriminate the studies based on which measure of loneliness they use, but we only included studies focusing on loneliness as opposed to related concepts such as social isolation;
8. We included only studies written in English.

The final pool of 109 articles included six meta-analyses, six reviews (four systematic), and 97 individual articles. Tables A3-A5in the Supplementary Materials show basic summaries of all these studies.

There are two final methodological points to make. In an effort to uncover causal effects, we isolated longitudinal studies (and some other designs, such as propensity score matching). However, these represent a minority of the evidence gathered, otherwise mostly coming from cross-sectional studies presenting correlational relationships. Second, while the most robust body of evidence on risk factors for loneliness is related to older adults, we made an effort to find research focusing also on other age groups (starting with adolescents).

## 3 Results

### 3.1 Individual factors

#### 3.1.1 Age

A measure of age is included in almost all studies, but often just as a control variable or a moderator variable. Nevertheless, a number of studies focus on changes in the prevalence of loneliness across life span.

Evidence from these is mixed. Some find elevated levels of loneliness among adolescents or young adults, lower levels in mid-adulthood, and increased levels again in old age (Lasgaard et al., 2016; Mund et al., 2020; Victor and Yang, 2012), that is, a U-shaped relationship between age and loneliness. Others find that loneliness decreases linearly with age (Barreto et al., 2021; Beutel et al., 2017; Tonković et al., 2021), and some that it increases (Hansen and Slagsvold, 2016; Hutten et al., 2022). The U-shaped relationship has been found also in some studies about specific age groups, for instance among students (18–35 years old) (Hysing et al., 2020) and middle-aged to older adults (aged 50 years or more) (Pinquart and Sörensen, 2001). This suggests there may be a more complex, non-linear relationship overall, with several peaks and dips in feelings of loneliness at different ages (the biggest peaks remaining at the young and old age) (Hawkley et al., 2020b; Luhmann and Hawkley, 2016).

The link between age and loneliness may depend on the context (i.e., a group of countries) (Hansen and Slagsvold, 2016; Tonković et al., 2021; Yang and Victor, 2011) and it also varies with how loneliness is measured (Nicolaisen and Thorsen, 2014), with sex (Yang and Victor, 2011), and their interplay (von Soest et al., 2018).

Nevertheless, the link between age and loneliness is often very weak (e.g., Hajek and König, 2020), and ceases to be statistically significant when other risk factors are considered (e.g., Dahlberg et al., 2021; Hawkley and Kocherginsky, 2018). This is confirmed by a meta-analysis of longitudinal studies on stability and changes in loneliness across life span, which finds that even though there seems to be a U-shaped relationship between age and loneliness cross-sectionally, the mean level of loneliness is quite stable across life span (Mund et al., 2020).

Hence, age is related to loneliness rather indirectly and differences in loneliness across the life span are plausibly due to varying prevalence of other loneliness risk factors across age groups. This is in line with the theoretical proposition that age is a distal factor of loneliness, operating through others.

#### 3.1.2 Sex

Sex is another factor that is present in almost every study, at least as a control variable. Sometimes, it is not included as a risk factor for loneliness, but allows other risk factors for loneliness to be studied separately for men and women. Such studies are not included in this section.

A recent meta-analysis looking at the relationship between sex and loneliness (Maes et al., 2019) concludes that men are slightly lonelier than women, but the difference is very small overall and often not significant (Maes et al., 2019). However, this meta-analysis has a design feature that may have substantially impacted the results – it focuses solely on studies that use standardised questionnaires to measure loneliness, which usually ask about loneliness only indirectly. When direct measures of loneliness are used instead, the prevalent finding is that women are lonelier than men or that there are no significant sex differences in loneliness (Bayat et al., 2021; Lykes and Kemmelmeier, 2013; Nicolaisen and Thorsen, 2014; von Soest et al., 2018). A possible reason for this discrepancy is a differing willingness to admit feeling lonely between men and women, with men being more likely to hide their feelings if asked directly (Borys and Perlman, 1985). If that is the case, a more accurate picture of sex differences in loneliness could be gained from the meta-analysis.

To make matters even more complex, findings related to sex also depend on what type of loneliness is measured. Men are more likely to be found more socially lonely, while emotional loneliness is found more in women (Nicolaisen and Thorsen, 2014; Penning et al., 2022; ten Kate et al., 2020; von Soest et al., 2018). However, it could again be the case of women being more willing to admit emotional loneliness, and men perceiving it as more acceptable to just admit social loneliness.

Age is potentially an important moderator of the sex-loneliness relationship, though there are discordant results on whether it strengthens or weakens it (Barreto et al., 2021; Franssen et al., 2020; Maes et al., 2019; Victor and Yang, 2012).

In summary, results on sex differences in loneliness are dependent on what type of loneliness is measured and how, possibly because men and women differ in their willingness to acknowledge feelings of loneliness. Nevertheless, it may also be that there is not a statistically significant relationship, meaning that sex, like age, only correlates with other factors that then impact loneliness directly. In other words, sex also seems to be a distal risk factor for loneliness.

#### 3.1.3 Race/ethnicity/migration background

The majority of studies focus on having a migration background (measured as nationality or an indicator of not being of a majority ethnicity), while fewer articles (all from the US) look at race, which, unlike migration background, is usually just added as a control variable.

The results on the impact of race on loneliness are mixed, with some research finding that people who identify as “white” are lonelier than others (Dahlberg et al., 2021; Hawkley and Kocherginsky, 2018; Hawkley et al., 2020b), other finding that they are less lonely compared to other races at least in older age (Warner and Kelley-Moore, 2012), and other yet revealing no statistically significant differences (Dahlberg et al., 2021; Greenfield and Russell, 2011; Shovestul et al., 2020; Warner and Kelley-Moore, 2012). Possibly, the link between race and loneliness is rather indirect (e.g., through differences in education or income), and hence may disappear when other factors are accounted for (Hawkley et al., 2008).

European studies looking at how migration background affects loneliness find a prevalently positive relationship; in other words, that migrants of all ages report higher levels of loneliness compared to native populations (e.g., Bayat et al., 2021; Buecker et al., 2021b; Franssen et al., 2020; Hysing et al., 2020; Niedzwiedz et al., 2016; Pagan, 2020), with some exceptions (Hutten et al., 2022; Marquez et al., 2022; Pan et al., 2023). However, in some studies this is true only unless other loneliness risk factors are taken into account, in which case the relationship flips sign (Fokkema and Naderi, 2013; ten Kate et al., 2020; Visser and El Fakiri, 2016). Suggested mediating factors that may cause this change are education and health-related outcomes (Visser and El Fakiri, 2016), or satisfaction with social relationships (ten Kate et al., 2020).

A possible explanation of different levels of loneliness between native and migrant populations is the feeling of belonging to or fitting one's environment, as suggested by the following findings: Older migrants in Canada who are of European (especially British and French) origin do not feel lonelier than native Canadians, but those of non-European origin do (de Jong Gierveld et al., 2015). Children in Denmark who share self-identified ethnicity with larger parts of their classrooms are less likely to feel lonely than those sharing it with smaller parts of their classroom (controlling for migration background) (Madsen et al., 2016). The number of local relatives and friends (and contact frequency with the latter) is linked to lower levels of loneliness among migrants in Belgium, while the frequency of transnational contact with friends and family is not (Koelet and de Valk, 2016).

In summary, both race and migration are possibly associated with loneliness rather indirectly (confirming their classification as distal factors for loneliness), with the channels of influence being either objective socio-economic circumstances, such as health or income, or subjective feelings of belonging.

#### 3.1.4 Education

We found no articles that study the relationship between loneliness and education specifically, but education is commonly used as a control variable to indicate socio-economic status, usually alongside income and employment status. Education is usually measured as a categorical variable and only a minority of studies measure it in years.

The evidence on the relationship between education and loneliness among older adults is quite mixed, as several literature reviews show (Cohen-Mansfield et al., 2016; Dahlberg et al., 2021). One meta-analysis finds a negative (i.e., protective) relationship (Pinquart and Sörensen, 2001), and some (but not all) very recent studies confirm this (Arpino et al., 2022; Guthmuller, 2022; Nyqvist et al., 2021; Rapolienė and Aartsen, 2022).

In some studies of general population, a higher level of education acts as a protective factor for loneliness (e.g., Hansen and Slagsvold, 2016; Hutten et al., 2022; Pagan, 2020). In some others, the negative relationship is found in bivariate analyses, but disappears when other relevant factors are controlled for (e.g., Dahlberg et al., 2018; Nicolaisen and Thorsen, 2014b; Tonković et al., 2021).

This would confirm the theoretical suggestion that education is linked to loneliness only indirectly, through other risk factors. In one study with middle-aged adults from the US, the negative relationship between having a high school diploma and loneliness is partially explained by chronic stress in everyday life, social network size, and relationship quality (Hawkley et al., 2008). In another one, conducted with a sample of the general population in Germany, it is income instead that explains this relationship (Luhmann and Hawkley, 2016). Moreover, when other covariates are added to the models in this study, the association between education and loneliness flips sign, suggesting that having accomplished more years of education is actually linked to more loneliness. Other single studies suggest that the link between loneliness and education depends on sex (Hansen and Slagsvold, 2016; Kung et al., 2022; Niedzwiedz et al., 2016), country of origin (Sundström et al., 2009; Visser and El Fakiri, 2016), or age (Franssen et al., 2020; Victor and Yang, 2012), even though the last two studies actually find discordant results.

Hence, the direct link between education and loneliness seems to be weak. Education is another one of the more distal (structural) factors of loneliness, being linked to it mostly through other factors, such as social networks or financial resources.

#### 3.1.5 Employment

A recent systematic review summarises evidence on the relationship between (un)employment and loneliness (Morrish and Medina-Lara, 2021). Most other studies use work status as one of the control variables, either binary (indicating being employed or not) or categorical (splitting people into those employed full-time, part-time or unemployed, eventually into those employed, retired, or having other occupations).

The systematic review shows that loneliness is lower for the employed and higher for the unemployed (Morrish and Medina-Lara, 2021). However, oftentimes these relationships fail to achieve statistical significance and sometimes even the opposite results are found (Luhmann and Hawkley, 2016).

It is possible that employment has a differential link with loneliness based on age and geographical area. Having a (full-time) job is associated with lower levels of loneliness compared to being without a job among middle-aged adults, but among younger adults, both those working full-time and not working at all have elevated loneliness levels (Hutten et al., 2022; Luhmann and Hawkley, 2016). Those aged less than average for a given life event react more strongly in terms of changes in loneliness to both transition into paid employment and job loss compared to those aged more than average (Buecker et al., 2021). Also, the link between being unemployed and lonely seems to be the strongest in the countries of continental (Western) Europe (Tonković et al., 2021).

Further research is needed to understand how different types of work arrangements are linked to loneliness. For now, there is only a hint of evidence from Belgium suggesting that temporary workers are lonelier than those with permanent contracts (Moens et al., 2021).

When looking at evidence from longitudinal studies, it looks like there is potentially a bidirectional relationship between employment and loneliness (Morrish and Medina-Lara, 2021). A study using propensity score matching and longitudinal design found an increase in loneliness in anticipation of the job loss and a slower decrease afterwards, an increase in loneliness after starting a paid employment, and interestingly also a decrease in loneliness after retirement (Buecker et al., 2021; the last result also found in Kung et al., 2022). However, the same study also found group differences in loneliness between those who experience a job loss during the study and the rest, suggesting that people who are lonelier may be more likely to experience a job loss.

To summarise, while cross-sectional evidence mostly points towards unemployment being linked to higher levels of loneliness (possibly being a more proximal factor of loneliness than others analysed so far, but still rather distal), there is not strong evidence proving that this relationship is causal. In fact, it is plausible that it is bidirectional.

#### 3.1.6 Financial situation

Studies on loneliness measure different concepts related to financial matters – most commonly it is household income, but some research focuses on household wealth or subjective perceptions of financial situation. Financial situation is mostly used as a control variable and only a few articles investigate the relationship between financial situation and loneliness specifically (Bosma et al., 2015; Hawkley et al., 2020; Niedzwiedz et al., 2016).

Longitudinal studies provide mixed results – some of them find that worse financial situation is linked to an increase in loneliness (Hajek and König, 2020; Kung et al., 2022; von Soest et al., 2018), while others do not find a statistically significant relationship (Dahlberg et al., 2021; Kristensen et al., 2021). One study trying to shed light on the issue of causality shows that a substantial negative income shock leads to increased subsequent feelings of loneliness, independently of baseline income levels (Hawkley et al., 2020). The relationship is not mediated by health indicators, relationship quality or church attendance. Wealth shocks, on the other hand, do not have a significant effect on subsequent loneliness.

A predominantly negative relationship between a better financial situation and loneliness has been confirmed by cross-sectional studies (e.g., Hawkley et al., 2020b; Hutten et al., 2022; Niedzwiedz et al., 2016; Rapolienė and Aartsen, 2022; Tonković et al., 2021), including a meta-analysis on loneliness in older adults (Pinquart and Sörensen, 2001). This relationship possibly depends on age and country of residence. Having enough financial resources is more important for loneliness in middle-aged adults compared to young or older adults (Luhmann and Hawkley, 2016; Pagan, 2020), and the link between financial imbalance and loneliness weakens with increasing age (Franssen et al., 2020). Income difficulties are linked to loneliness in older adults more strongly in Eastern rather than Western Europe (de Jong Gierveld et al., 2012), and limited socio-economic resources together with poor health can explain higher levels of loneliness among older adults in Spain, Italy, Czech Republic, and Poland (Fokkema et al., 2012). Some studies also found that the relationship between financial situation and loneliness is moderated by health, and social network and participation indicators (Bosma et al., 2015; Hawkley et al., 2008; Marquez et al., 2022; Niedzwiedz et al., 2016; Nyqvist et al., 2021; Smale et al., 2022).

To sum up, a worsened financial situation plausibly leads to increased feelings of loneliness, especially in middle-aged adults and older adults from Eastern Europe. Possibly, a part of the relationship is due to other factors, such as health and social participation, suggesting that financial situation is a rather distal risk factor for loneliness.

#### 3.1.7 Psychological factors

The main psychological factor that stood out as a potential risk factor for loneliness were the Big Five personality traits, which include extroversion, neuroticism (i.e., a long-term tendency to experience negative emotions), agreeableness, openness, and conscientiousness. There is even a meta-analysis available on this topic (Buecker et al., 2020), which will form the basis of this section. The rest of the studies that we found focus on many distinct concepts (e.g., negative affect, shyness, low mood, personal mastery, feelings of uselessness, irritability, hopelessness, resilience, optimism), so the evidence on each phenomenon often comes from a very low number of studies, preventing any robust conclusions. These other findings will be mentioned more as examples and should be taken with appropriate caution.

The meta-analysis showed that when looking at the relationships between loneliness and each of the five traits individually, extroversion and neuroticism are linked to loneliness most strongly, reaching a large effect size (Buecker et al., 2020). The link between loneliness and neuroticism is also the only positive one, meaning that higher levels of neuroticism are linked to higher levels of loneliness. All the other associations are negative. Agreeableness and conscientiousness both have a moderately strong association with loneliness, while openness is linked to loneliness only weakly. When controlling for the other four personality traits, the relationship between openness and loneliness loses its statistical significance, and all other relationships weaken.

The above results, suggesting that mostly neuroticism (or its opposite, emotional stability) and extroversion are associated with loneliness (and possibly its onset), have been broadly confirmed by other single studies published in the most recent years (Abdellaoui et al., 2019; Altschul et al., 2021; Guthmuller, 2022; Stavrova et al., 2022; von Soest et al., 2018), with some exceptions (Lampraki et al., 2019; Ormstad et al., 2020). The link between neuroticism and loneliness has been examined also genetically and by studying twins in the Netherlands, and the results suggest it is bidirectional, with a stronger influence of neuroticism on loneliness than the other way around (Abdellaoui et al., 2019).

Other psychological characteristics found to be associated with loneliness are for instance low self-esteem or self-efficacy, both in adolescents and older adults (Cohen-Mansfield et al., 2016; Mahon et al., 2006), shyness in adolescents (Mahon et al., 2006), low levels of mastery in older adults (Ejlskov et al., 2017; van Tilburg, 2021), high negative affect (possibly through a bidirectional relationship) (Böger and Huxhold, 2018) or low self-control (Stavrova et al., 2022).

Nevertheless, extroversion and neuroticism remain the two psychological factors whose link with loneliness has the strongest empirical support up to date. Further research will be needed to be able to draw robust conclusions about other psychological factors and to determine their place in the “distal-proximal” factors theoretical framework.

#### 3.1.8 Health

The literature on the relationship between health and loneliness adopts two different approaches – it examines the effects of loneliness on health (e.g., Baarck and Kovacic, 2022; Holt-Lunstad et al., 2015) or the effects of health on loneliness instead. Here, we focus on the latter, but the potential bidirectionality of this relationship should always be borne in mind, especially because many of the studies are cross-sectional.

Measures of physical or mental health are usually present in studies examining multiple risk factors for loneliness. Physical health is either measured objectively, reflecting chronic health conditions, disabilities, or functional limitations, or subjectively, as people's perception of their own health. Often, both types of measures are used within the same study. Mental health is usually assessed through established scales measuring depression or anxiety.

The review studies of loneliness in older adults mostly find that those with better subjective health and less functional limitations report feeling less lonely, even though statistical significance is not always reached (Cohen-Mansfield et al., 2016; Dahlberg et al., 2021; Pinquart and Sörensen, 2001).

Some longitudinal studies provide causal evidence of the effect of poor physical health on loneliness in middle-aged and older adults (Aartsen and Jylhä, 2011; Böger and Huxhold, 2018; Hajek and König, 2020; Hawkley and Kocherginsky, 2018), while some fail to find a significant effect (Nicolaisen and Thorsen, 2014b; Nyqvist et al., 2021). In one of the studies the link between physical health and loneliness vanishes when other risk factors, such as loss of a partner and reduced social activity, are considered (Aartsen and Jylhä, 2011). This suggests that social activity may be a mechanism through which health impacts loneliness. Other possible mediators include negative affect (Böger and Huxhold, 2018) or neuroticism (von Soest et al., 2018).

The prevalently positive relationship between bad physical health and loneliness is confirmed by cross-sectional studies and studies focusing on general population (e.g., Dahlberg et al., 2018; Hawkley et al., 2020b; Nicolaisen and Thorsen, 2014; Pagan, 2020; Warner and Kelley-Moore, 2012). Sometimes, whether a relationship is found or not depends on how exactly health is assessed (Ejlskov et al., 2017; Fokkema and Naderi, 2013; Marquez et al., 2022), on age (Franssen et al., 2020; Hutten et al., 2022; Victor and Yang, 2012) or geographical area (Nyqvist et al., 2019; Sundström et al., 2009).

When it comes to mental health, literature reviews and meta-analyses show that depression is often statistically significantly linked to loneliness in older adults (Cohen-Mansfield et al., 2016; Dahlberg et al., 2021) and also in adolescents, for which this link corresponds to a large effect size (Mahon et al., 2006). There is also some longitudinal evidence that an increase in depressive symptoms is associated with an increase in feeling lonely among middle-aged and older adults (Hajek and König, 2020; Nyqvist et al., 2021).

A strong association between mental health and loneliness is found also in cross-sectional studies with samples of all age categories, with mental health sometimes being among the strongest correlates of loneliness (e.g., Franssen et al., 2020; Guthmuller, 2022; Hutten et al., 2022; Lodder et al., 2015; Victor and Yang, 2012).

In summary, there is evidence that physical and mental health are associated with loneliness. While some evidence is longitudinal, suggesting that worse health may be causing loneliness, there is evidence also for the contrary – loneliness causing worse health (Baarck and Kovacic, 2022). Thus, it is fair to conclude that the relationship between health and loneliness is bidirectional. In terms of the “distal-proximal” factors theoretical framework, health seems fall into the latter category, even though the link of physical health to loneliness may be mediated through social activity or psychological factors.

#### 3.1.9 Marital/partner status

Marital/partner status is normally used either as a categorical variable (with the most common categories being married or partnered, divorced or separated, widowed, and single or never married) or as a binary variable. There is also research that examines the quality of the relationship with the partner or spouse, on top of simply having one.

In cross-sectional studies, having a partner or a spouse is consistently linked to lower levels of loneliness, while being single, divorced or widowed are associated with higher levels of loneliness (e.g., Arpino et al., 2022; Cohen-Mansfield et al., 2016; Guthmuller, 2022; Nicolaisen and Thorsen, 2014b; von Soest et al., 2018). This relationship with loneliness is often found to be one of the strongest among all predictors, especially when it comes to widowhood (Fokkema et al., 2012; Hajek and König, 2020; Lykes and Kemmelmeier, 2013; Tapia-Muñoz et al., 2022).

Longitudinal studies broadly confirm the above and suggest that the relationship between partner status and loneliness may be causal. Losing a partner leads to increased loneliness at a later date among middle-aged and older adults (Aartsen and Jylhä, 2011; Dahlberg et al., 2021; Nicolaisen and Thorsen, 2014b; Nyqvist et al., 2021).

Partner or marital status exert a different influence on loneliness depending on age and sex. Partner status is not significantly related to loneliness in the youngest adults (<30 years old) (Franssen et al., 2020; Luhmann and Hawkley, 2016; Victor and Yang, 2012), and it impacts loneliness the most in young to middle-aged adults (31–60–65 years old) (Luhmann and Hawkley, 2016; Victor and Yang, 2012). Also, partner status matters more for men's rather than women's loneliness, regardless of their age (Hysing et al., 2020; Kung et al., 2022; Pagan, 2020; van den Broek and Grundy, 2017).

There is some evidence that loneliness increases already before a divorce or a separation, while no such change is observed for widowhood, where the change in loneliness happens only after the event (Buecker et al., 2021). Speculatively, this could suggest that the quality of the relationship with the partner plays a role in feeling lonely. It is easy to imagine that separation and divorce are preceded by a period of worsening of the relationship, which could explain the increase in loneliness.

In fact, this speculation is confirmed by scientific evidence. Good marital/partner relationship, emotional support received from the partner, having the partner as a close confidant or even good sex life are all protective factors for loneliness, while bad quality relationship with the partner is associated with more feelings of loneliness (de Jong Gierveld et al., 2009; Fokkema and Naderi, 2013; Hawkley et al., 2008; Warner and Kelley-Moore, 2012).

To conclude, partner status (or its change) is one of the most important direct (proximal) determinants of loneliness. The group especially at risk of feeling lonely are widows, but also people who are divorced or single. However, it is not just the fact of having a partner that affects loneliness, but also the quality of the relationship with this partner, so even partnered or married adults are at some risk of feeling lonely.

#### 3.1.10 Living arrangements

Mostly, living with other people (measured as household size, an indicator of living alone or a detailed account of different living arrangements) is included in studies as a control variable, with a few articles examining this issue specifically (mainly among older adults). Literature focusing on older adults also examines the impact of living with children or in residential care/nursing homes. Almost all studies on living arrangements and loneliness are cross-sectional.

Among the few longitudinal studies assessing the impact of a change in living arrangements (living alone, transition to an “empty nest”, starting cohabitation) on subsequent loneliness, not one finds statistically significant effects (Buecker et al., 2021; Hawkley and Kocherginsky, 2018; Kristensen et al., 2021). However, one of these studies finds group differences in loneliness between those who started cohabiting during the study and their propensity score-matched counterparts who did not (Buecker et al., 2021). This could mean that there is a reverse relationship between loneliness and living arrangements; that is, lonelier people may be more likely to live alone.

In any case, in cross-sectional studies, living alone is consistently shown to be associated with significantly higher levels of loneliness compared to other living arrangements and it is often one of the strongest correlates (e.g., Altschul et al., 2021; Dahlberg et al., 2022; Hansen and Slagsvold, 2016; Lykes and Kemmelmeier, 2013; Nyqvist et al., 2019; Sundström et al., 2009; Tonković et al., 2021). Digging deeper, it looks like the presence of a partner in the household makes the biggest difference for loneliness, at least for older adults (de Jong Gierveld et al., 2012; Greenfield and Russell, 2011; Hansen and Slagsvold, 2016). On the other hand, living with children (or parents in case of young adults) does not seem to help protect from loneliness (Hysing et al., 2020; Hawkley et al., 2020b; Luhmann and Hawkley, 2016). In fact, there may be a U-shaped relationship between household size and loneliness, with older adults living with exactly one other person being the least lonely (Dahlberg et al., 2022).

Even though living arrangements are quite a universal correlate of loneliness, there is evidence on some interaction of their effect with sex and age. Living with someone else offers a stronger protection from loneliness to men than women (Altschul et al., 2021; Hansen and Slagsvold, 2016; Hysing et al., 2020). Also, living alone seems to be less important for loneliness among young adults compared to other age groups (Hutten et al., 2022).

As for older adults living in residential care/nursing homes, evidence from literature reviews and a meta-analysis suggests that this is linked to higher levels of loneliness compared to living in the community (Cohen-Mansfield et al., 2016; Dahlberg et al., 2021; Pinquart and Sörensen, 2001), even though the differences are not always statistically significant (Lyu and Forsyth, 2021).

In summary, living alone is strongly associated with more feelings of loneliness and living with others is linked to less loneliness, especially if there is a partner in the household. In case of old adults, living in nursing homes or residential care is possibly associated with higher levels of loneliness than community living. Living arrangements seem to be one of the more proximal factors of loneliness, possibly operating through their impact on an individual's social network. Nevertheless, robust evidence on causal relationships is lacking for now.

#### 3.1.11 Social network

The most common measured aspects of personal social networks are frequency of contact with different figures (family, friends, neighbours), social network size, quality of social relationships, social support, and also presence of an intimate figure in one's life (and their identity). Usually, multiple measures are used in the same study.

More often than not, larger social network size and the presence of at least one close, intimate figure (a confidant) are linked to less feelings of loneliness (e.g., de Jong Gierveld et al., 2015; Guthmuller, 2022; Lykes and Kemmelmeier, 2013; Milicev et al., 2022; Nyqvist et al., 2019; Smale et al., 2022). However, longitudinal studies often fail to find statistically significant effects, at least when it comes to older adults (Dahlberg et al., 2021; Nyqvist et al., 2021). Possibly, loneliness also influences the size of the social network, and this link is stronger than vice versa (Böger and Huxhold, 2018).

Perhaps more important than the number of social contacts is the frequency of socialising with them. The evidence shows quite consistently that the more frequently people socialise with others, the less lonely they are (e.g., Ejlskov et al., 2017; Franssen et al., 2020; Guthmuller, 2022; Pinquart and Sörensen, 2001; Victor and Yang, 2012). There is also a hint from longitudinal studies that meeting socially more frequently leads to steeper declines in loneliness (Hawkley and Kocherginsky, 2018; von Soest et al., 2018), even though it is not always the case (Nyqvist et al., 2021). The strength of the relationship depends on who one socialises with and how. Contact with friends is generally more protective than contact with family and neighbours (de Jong Gierveld et al., 2015; Franssen et al., 2020; Hutten et al., 2022; Koelet and de Valk, 2016; Nyqvist et al., 2016; Pinquart and Sörensen, 2001), but sometimes it is the relatives who matter the most (Hawkley et al., 2020b; ten Kate et al., 2020), with the difference being possibly due to different cultural settings (Lykes and Kemmelmeier, 2013). Also, while local contacts are related to less loneliness, contact with people abroad is not (Koelet and de Valk, 2016; Luhmann and Hawkley, 2016). Similarly, going out with friends when one feels like it is associated with less loneliness in young people (Marquez et al., 2022), but the amount of time spent interacting with them on social media is not (Marquez et al., 2022; Milicev et al., 2022). Also, when satisfaction with the contact with friends is controlled for, the statistically significant link between objectively measured frequency of contact and loneliness disappears (Nicolaisen and Thorsen, 2017). In other words, what seems to matter for loneliness is being satisfied with how often one sees friends, regardless of how often it actually is.

Related to that is the issue of relationship quality. According to a meta-analysis of studies on loneliness among older adults, a better quality of relationships is associated with lower levels of loneliness and it matters more than the quantity of these relationships (Pinquart and Sörensen, 2001). Also for children and adolescents, good friendship quality is associated with less loneliness, and the link is possibly stronger than that of friendship quantity (Lodder et al., 2015). Moreover, a recent meta-analysis shows that the relationships between both friendship quantity and quality and loneliness could be bidirectional (Schwartz-Mette et al., 2020). Bad relationships in family are linked to enhanced feelings of loneliness among adolescents (Heshmati et al., 2021; Yang et al., 2020), as well as the fact of being bullied (Bayat et al., 2021; Yang et al., 2020). Adverse conditions in childhood may even impact loneliness later in life (Guthmuller, 2022; Nicolaisen and Thorsen, 2014b). Conflicts in personal relations in general are strongly linked to feelings of loneliness (Tonković et al., 2021), while good relationships, for instance with neighbours, seem to protect from loneliness (Buecker et al., 2021b) and so does getting social or emotional support from one's social network (Dahlberg et al., 2018; Hawkley and Kocherginsky, 2018; Krause, 2016; Rote et al., 2013). Similar associations between social and emotional support and loneliness have been found for both adolescents (Mahon et al., 2006; Yang et al., 2020) and older adults (especially if the support comes from family members) (Cohen-Mansfield et al., 2016; Dahlberg et al., 2018; de Jong Gierveld et al., 2009; Hawkley and Kocherginsky, 2018). Interestingly, also giving emotional or instrumental support seems to be associated with less loneliness among older adults (de Jong Gierveld et al., 2009, 2012), and in some cases receiving the support actually has the opposite effect (de Jong Gierveld et al., 2012; Fokkema et al., 2012). Similarly, providing non-burdensome informal care is associated with less loneliness across age categories, while the relationship reverses if the care is burdensome (Hutten et al., 2022).

A specific issue is having (more) children, which seems to be associated with lower levels of loneliness among older adults, but this association possibly depends on sex, partner status or type of loneliness measured (Arpino et al., 2022; Fokkema et al., 2012; Hansen and Slagsvold, 2016; Penning et al., 2022) and is not always statistically significant, especially in studies including also other age groups (Lampraki et al., 2019; Lasgaard et al., 2016; Pan et al., 2023; von Soest et al., 2018). There is also evidence that more than the mere fact of having children, it is the relationship quality and frequency of contact with them that matter (de Jong Gierveld et al., 2009; Niedzwiedz et al., 2016).

To summarise, loneliness is indeed strongly and directly linked to different aspects of people's social networks and it seems that more than the size of the network, it is its quality and functioning that define this link. Frequent contact with friends and family, good relationships with them, and receiving and giving social and emotional support are all important protective factors against feelings of loneliness.

#### 3.1.12 Social activity

Social activity is usually measured by the frequency of participating either in a mix of activities (producing an overall measure of engagement) or in specific activities, such as religious activities or voluntary work. Some studies also look at the effect of mere membership in different groups or organisations.

In general, studies among older adults tend to find a positive link between the lack of social activity and loneliness (Fokkema et al., 2012; Niedzwiedz et al., 2016). In other words, the less a person is socially engaged, the more likely they are to feel lonely. Also, there is some evidence for this relationship to be due to a reduction in social activity (Aartsen and Jylhä, 2011) or to the dissatisfaction with the amount of social activity rather than its absolute level (Guthmuller, 2022).

In the general population, also the relative level of social activity with respect to others of the same age has a significant (even though small in size) link with loneliness (Lykes and Kemmelmeier, 2013; Victor and Yang, 2012). Moreover, there is some evidence of the bidirectionality of the relationship, with loneliness having a stronger impact on social activity than the other way around (Böger and Huxhold, 2018).

When looking at frequency of engaging in specific social activities (such as voluntary work, religious activities, or various group meetings) across age groups, the association with loneliness is usually not statistically significant, at least when other factors are considered (e.g., Hawkley and Kocherginsky, 2018; Hutten et al., 2022; Krause, 2016; Luhmann and Hawkley, 2016; MacDonald et al., 2020; Marquez et al., 2022), or it is so only in some countries (Tonković et al., 2021). A mere membership in groups or organisations does not seem to correlate with loneliness of older adults significantly (de Jong Gierveld et al., 2015; Hawkley et al., 2008; Nyqvist et al., 2021), or if so, just for specific subgroups (Lampraki et al., 2019).

Possibly, social activity affects loneliness through its impact on social networks. It has been found that social support accounts for the association between religious attendance and loneliness (Rote et al., 2013), while the link between group membership and meetings on loneliness has been explained by social network size and frequency of contact with others (Hawkley et al., 2008; Rote et al., 2013).

To sum up, being more socially engaged in general is associated with lower levels of loneliness. Nevertheless, the relationship is possibly bidirectional and mediated by social network characteristics, making social activity a proximal, but not a completely direct risk factor for loneliness.

### 3.2 Societal factors

#### 3.2.1 Environment

Relatively few studies focus on the relationship between the living environment and loneliness, and also, few use the environmental characteristics as control variables. Almost all evidence is cross-sectional. The ways of measuring the characteristics of the environment vary between studies – it can be for instance the urban/rural divide, population or residential density, neighbourhood characteristics, such as access to different facilities, or subjective perceptions of one's neighbourhood.

Recently, three reviews were published relating the environmental characteristics to feelings of loneliness. Two of these focus on the built environment (among older adults (Lyu and Forsyth, 2021) and in the general population (Bower et al., 2023)), while the third one on the green spaces only (Astell-Burt et al., 2022). Therefore, we base our discussion mainly on these reviews and complement it with some other interesting findings from individual studies.

A better availability and accessibility of facilities in the neighbourhood (in terms of e.g., services, social sites, or leisure-time facilities) are mostly linked to lower levels of loneliness (Bower et al., 2023; Buecker et al., 2021b; Lyu and Forsyth, 2021), and the same holds for access to green spaces, even if the link is not always statistically significant (Astell-Burt et al., 2022; Bower et al., 2023; Buecker et al., 2021b; Lyu and Forsyth, 2021). A better walkability of the neighbourhood (especially when measured subjectively) is also mostly linked to less loneliness in older adults, but again, sometimes without a statistically significant link (Lyu and Forsyth, 2021). In addition, overall satisfaction with the neighbourhood or its quality are associated with less loneliness and so is the feeling of belonging to the local community or neighbourhood (Bower et al., 2023; de Jong Gierveld et al., 2015; Lyu and Forsyth, 2021, Milicev et al., 2022; Nyqvist et al., 2016; Smale et al., 2022; Visser and El Fakiri, 2016).

Residential density, degree of urbanisation, or living in an urban or a rural area do not seem to have a significant association with loneliness, at least as long as other built environment characteristics are accounted for (Bower et al., 2023; Guthmuller, 2022; Lyu and Forsyth, 2021; Milicev et al., 2022), with some exceptions showing that a higher degree of urbanisation is linked to somewhat higher levels of loneliness (Lykes and Kemmelmeier, 2013; MacDonald et al., 2020). As for housing or residential density in particular, some studies find no significant link to loneliness (Bower et al., 2023) and others find that there is a relationship but it depends on the type of housing and may be mediated by demographic and socio-economic characteristics (Bower et al., 2023; Lai et al., 2021). When it comes to transportation, there seems to be a negative link to loneliness, especially looking at public transport (Lyu and Forsyth, 2021), possibly thanks to facilitating access to public spaces and interactions (Bower et al., 2023).

In summary, certain characteristics of an individual's living environment show rather strong associations with feelings of loneliness, while others do not. It is mostly the subjective evaluation of the neighbourhood and access to different facilities or green spaces that matter for loneliness. Arguably, these characteristics influence loneliness only indirectly, through a better sense of belonging in the neighbourhood and easiness of socialising with other local inhabitants.

#### 3.2.2 Contextual factors

Two main channels of influence of the context on loneliness have been proposed on theoretical level: (i) country-level socio-economic characteristics that, similarly to the local environment, shape the social lives of people, and (ii) socio-cultural context that affects individual expectations and subjective evaluations of social relations (de Jong Gierveld and Tesch-Römer, 2012). In explaining the potential cross-country differences in loneliness, another channel exists, which is the socio-demographic composition of the population (Dykstra, 2009). It is important to note that the theoretical frameworks which have been created to explain cross-country differences in loneliness specifically focus on older adults in Europe. We would argue that these frameworks can be used as well for other age groups in Europe, but their usage in other geographical contexts may not be as appropriate.

It seems that very little research has been dedicated to studying contextual factors of loneliness empirically. Some studies just report cross-country differences and mention contextual factors as a potential explanation. Such studies from Europe consistently find a North-West vs South-East divide in loneliness in some form, with people from the latter group of countries experiencing higher levels of loneliness, on average (D’Hombres et al., 2018; Fokkema et al., 2012; Hansen and Slagsvold, 2016; Sundström et al., 2009; Yang and Victor, 2011). In line with the above (Dykstra, 2009), we explore the empirical evidence for the three possible explanations of this divide.

The first is population composition, i.e., the idea that loneliness differs between countries because these differ in prevalence of certain individual characteristics that are linked to loneliness. Some empirical evidence shows that more people with poor health, limited socio-economic resources, and more unmarried individuals can explain higher levels of loneliness in some Eastern and Southern European countries (Fokkema et al., 2012; Hansen and Slagsvold, 2016; Rapolienė and Aartsen, 2022). Similarly, higher levels of loneliness among older adults in the European post-totalitarian countries can be explained through lower levels of social engagement, which is in turn due to lower levels of trust (Rapolienė and Aartsen, 2022).

Another possibility is that differences at the country level account for varying levels of loneliness in Europe. For example, loneliness among older adults seems to be more likely in countries with higher income inequality, even after controlling for individual-level factors (Tapia-Muñoz et al., 2022). Also, welfare systems in Europe are potentially associated with levels of loneliness among older adults. In particular, the absence of loneliness is more likely in the Nordic, Anglo-Saxon, and Continental welfare regimes, and less likely in the Southern and Eastern regimes (Nyqvist et al., 2019). A link between country secularism and loneliness has also been examined, but without finding empirical support (Das, 2022). It is plausible that both income inequality and welfare systems are linked to loneliness only indirectly, by shaping other risk factors that then impact feelings of loneliness more directly (de Jong Gierveld and Tesch-Römer, 2012).

The final proposed explanation is that the individual and country factors interact, so that the same situation is perceived differently in different countries, based on their culture and prevalent social norms. For instance, loneliness may be higher in countries that are collectivist, because people may have higher expectations about social relations (e.g., that old parents live with their children) and if these are not fulfilled (e.g., an old person ends up living alone), the suffering may be substantial (de Jong Gierveld and Tesch-Römer, 2012; Dykstra, 2009). In more individualistic countries, the same outcome (i.e., an old person living alone) may be seen as appropriate and hence not necessarily cause feelings of loneliness. It could still be, however, that loneliness is higher in individualistic countries, but for different reasons (Heu et al., 2021). For instance, living alone has been shown to be a strong risk factor for loneliness and thus is likely to matter whether it is in accordance with the prevalent social norm or not. Research empirically testing the impact of social norms and culture is almost inexistent and does not clearly support either of the previous propositions. One study from Europe suggests that people in individualistic countries are somewhat less lonely (Lykes and Kemmelmeier, 2013), while another study conducted with non-representative samples of people from all over the world (though with a heavy overrepresentation of UK citizens) finds the opposite (Barreto et al., 2021).

Therefore, no empirically supported robust conclusions about the effects of culture or other country-level characteristics on loneliness can be drawn for the time being.

### 3.3 Risk factors during COVID-19

Studies on loneliness during the COVID-19 pandemic differ in several ways from the ones published before. There is less research focusing on risk factors (and more focusing on changes in loneliness) and there are only a few studies looking at how risk factors for loneliness changed compared to before the pandemic. Convenience samples of population (for instance recruited via social media) are relatively more common, but also, the focus of the research shifted towards loneliness in the general population rather than just among older adults.

Broadly speaking, the risk factors for loneliness during the COVID-19 pandemic were similar to those before the pandemic, but there were a number of interesting differences. While in pre-pandemic times, various shapes of the relationship between age and loneliness had been proposed, the prevalent finding during the pandemic was that the relationship was negative – loneliness and its increase were the highest among young adults and decreased with increasing age (e.g., Entringer and Gosling, 2022; Ikizer et al., 2022; Li and Wang, 2020; Lim et al., 2022; Wickens et al., 2021). In fact, the share of people aged 18 to 25 reporting feeling lonely during the first months of the pandemic almost quadrupled (Baarck et al., 2021). In general, the relationship between age and loneliness seemed stronger than before (Bu et al., 2020).

The geography of loneliness in Europe also changed. Before the pandemic, Northern European countries would display a significantly lower incidence of loneliness than the rest of the EU member states (Baarck et al., 2021). With the onset of COVID-19, the incidence increased everywhere to reach broadly similar levels (Baarck et al., 2021).

The evidence on the association between sex and loneliness had been quite mixed before the pandemic, depending on the measure used and type of loneliness measured. Finding emotional loneliness higher in women and social loneliness higher in men was common both before and during the pandemic (Bonsaken et al., 2021; van Tilburg et al., 2021). During the pandemic, however, most studies found that women were lonelier or experienced higher increases in loneliness than men (Entringer and Gosling, 2022; Lepinteur et al., 2022) with both direct (Arpino et al., 2022; Li and Wang, 2020; Wickens et al., 2021) and indirect measures (Bu et al., 2020b; Hoffart et al., 2020; Ikizer et al., 2022; Lin, 2023). Speculatively, this could be driven by non-randomness of the samples, which included a higher proportion of women willing to participate and possibly admit their feelings of loneliness.

The relationship between employment and loneliness seemed to be more consistently statistically significant during the pandemic than before, with studies finding that being unemployed was linked to higher levels of or increases in loneliness (Bu et al., 2020; Hoffart et al., 2020; Li and Wang, 2020; Lim et al., 2022; Völker, 2023), possibly when the unemployment was related to the pandemic itself (Lin, 2023). Interestingly, we found no evidence that those working from home experienced higher levels of loneliness compared to those with no change in their work setting (Entringer and Gosling, 2022; Ruffolo et al., 2021; Wickens et al., 2021).

In accordance with pre-pandemic times, living with a partner was consistently found to be a protective factor against loneliness (Arpino et al., 2022; Bonsaken et al., 2021; Li and Wang, 2020; van Tilburg, 2021), as well as receiving more social support (Bu et al., 2020b; Groarke et al., 2020), having larger social networks (Bu et al., 2020b; Lin, 2023; Milicev et al., 2022) or more frequent social interactions (Lampraki et al., 2022), with face-to-face interactions possibly being more protective than the virtual ones (Caro et al., 2022; Hoffart et al., 2022), which may have even backfired (Lampraki et al., 2022; Rumas et al., 2021). Living alone (Bu et al., 2020, 2020b; Choi et al., 2022; Lim et al., 2022; Wickens et al., 2021) and being divorced, widowed or single (Groarke et al., 2020; Hoffart et al., 2020; Wickens et al., 2021; Wilson-Genderson et al., 2021) remained important risk factors for loneliness.

Worse mental and physical health were other factors associated with higher loneliness or its increase during the COVID-19 pandemic (e.g., Caro et al., 2022; Groarke et al., 2020; Hu and Gutman, 2021; Lampraki et al., 2022; Völker, 2023; Wilson-Genderson et al., 2021).

Among the Big Five personality traits, neuroticism was the most strongly correlated with feelings of loneliness both before and during the pandemic (Ikizer et al., 2022). Neuroticism, but also extroversion, and conscientiousness were strongly related to increases in loneliness during the pandemic (Entringer and Gosling, 2022).

The findings on the relationship between income and loneliness during the pandemic were mixed (Entringer and Gosling, 2022; Groarke et al., 2020; Lim et al., 2022; Völker, 2023; Wickens et al., 2021), while other factors like education, ethnicity, or rural-urban divide were mostly found to be unrelated to loneliness or its changes, just as before the pandemic (e.g., Bu et al., 2020b; Choi et al., 2022; Groarke et al., 2020; Milicev et al., 2022; Rumas et al., 2021; van Tilburg, 2021).

Some studies also looked at possible risk factors specifically related to the pandemic. Among those, it was mostly feelings of worry (about different things) that were associated with increased levels of loneliness (Bonsaken et al., 2021; Hoffart et al., 2020, 2022; van Tilburg et al., 2021).

A general conclusion based on these findings is that mostly similar groups of people were at risk of being lonely during the COVID-19 pandemic as before, except for the young people who suffered important increases in loneliness.

## 4 Conclusions

In this narrative literature review, we summarised empirical evidence on risk factors for loneliness. Following the theoretical frameworks (de Jong Gierveld and Tesch-Römer, 2012; Hawkley et al., 2008), we examined distal, structural, and proximal individual-level determinants of loneliness, as well as societal factors. Our discussion was based on recent evidence from Europe and North America, covering the whole age range of the population where possible. The main results about each risk factor are summarised in Table A2in the Supplementary Materials.

Overall, several important findings stem from this review. First, the strength of the link between various risk factors and loneliness differs quite substantially. Broadly speaking, the theoretical categorisation into distal, structural, and proximal factors holds. Demographic factors are correlated with loneliness, but the link is often weak and becomes negligible if other factors are accounted for. Similarly, the association between socio-economic factors and loneliness is partially explained by more proximal factors. In general, however, lower socio-economic status is linked to higher levels of loneliness, possibly as a result of a bidirectional relationship. Some personality traits are linked to loneliness as well, such as extroversion or neuroticism. Loneliness is also related to the environment in which one lives, and possibly the socio-economic and socio-cultural context. However, what it all boils down to in the end is partner or marital status, living arrangements, and social network characteristics. These factors are all consistently found to be among the strongest predictors of loneliness. People who lost their partner as a result of death, divorce, or separation, who were never married, those living alone, not often in touch with friends or family, or lacking adequate social support, are all vulnerable to feelings of loneliness and the related consequences.

These findings should be interpreted bearing in mind the nature of the current review. It is a narrative review of quantitative research on loneliness, thus comes with all advantages and shortcomings of such a format. On one hand, it offers a well-structured overview of the most recent empirical evidence on risk factors for loneliness, understandable to a wide range of audiences. On the other hand, it does not offer any quantitative insights into the size of the effects found in the literature. Also, it only includes studies selected based on a set of specific criteria, which may have caused exclusion of some potentially relevant studies. Future research should focus on conducting meta-analyses of the risk factors for loneliness in order to get the quantitative insights missing here. The current review could then serve as a basis for inclusion and categorisation of risk factors.

With regards to policy implications of this review, an important insight is that many of the determinants of loneliness are interrelated – the association of some of the risk factors with loneliness seems to be shaped by other risk factors. Income, for instance, has a stronger link with loneliness among middle-aged adults than in other age groups. Marriage, partnership, and sharing a household with someone provide more protection against loneliness for men than for women. Each person is defined by a whole set of characteristics, each of which can be related to loneliness in a different way. When designing policies to combat loneliness, this complexity needs to be kept in mind. It may be that addressing individual risk factors in isolation will not be enough to tackle loneliness, and that more holistic solutions will be needed.

Finally, causal evidence about the risk factors for loneliness remains scarce. The large majority of existing studies are cross-sectional, only drawing associations between variables. Studies based on longitudinal data and using appropriate, robust methods (e.g., quasi-experimental designs, such as regression discontinuity, difference-in-differences or instrumental variables approach) that could prove causal relationships are often lacking. Future research will need to address this gap. In the meantime, scholars, policymakers, and practitioners reading this brief overview of a very rich literature should bear this caveat in mind and interpret the findings accordingly.

## Declaration of competing interest

The work of Martina Barjaková has been funded by the 10.13039/501100000780European Commission. This study is part of a broader project on loneliness in Europe carried out by the European Commission Directorate-General for Employment, Social Affairs & Inclusion (DG EMPL), in collaboration with the 10.13039/501100000900Joint Research Centre (JRC), upon request of the European Parliament. For more material and information, please visit the project webpage https://knowledge4policy.ec.europa.eu/projects-activities/loneliness-european-union_en.

## Data Availability

No data was used for the research described in the article.
