# Supplementary material for: Risk factors for loneliness: A literature review
Source: Soc Sci Med. 2023 Oct;334:116163. doi: 10.1016/j.socscimed.2023.116163 (PMC10523154; doi:10.1016/j.socscimed.2023.116163)
Supplement: Multimedia component 1 [file mmc1.docx]

**Table A1**

*Search Keywords*

| **Risk factor** | **Search keywords** |
| --- | --- |
| Age | *“age”, “life span”* |
| Sex | *“gender”, “sex”* |
| Race/ethnicity/migration background | *“migration”, “immigration”, “migrant”, “ethnicity”, “race”* |
| Education | *“education”* |
| Employment | *“unemployment”, “employment”, “work”* |
| Financial situation | *“income”, “financial”, “poverty”, “poor”, “wealth”, “socioeconomic”* |
| Psychological factors | *“psychological”* |
| Marital/partner status | *“partner”, “spouse”, “marital”, “marriage”, “widow”, “divorce”* |
| Living arrangements | *“living alone”, “living arrangements”, “household”* |
| Social network | *“social network”, “social contact”, “social support”, “relationship quality”, “friends”, “family”* |
| Social activity | *“social activity”, “engagement”* |
| Environment | *“urban”, “rural”, “environment”, “place”, “area”, “neighbourhood”* |
| Contextual factors | *“demography”, “welfare”, “inequality(ies)”, “culture”, “social norms”* |
| COVID-19 | *“covid”, “pandemic”* |

*Note:* All the keywords listed in this table were used together with the term “loneliness” in the search.

**Table A2**

*Summary of the evidence*

| **Factor** | **Summary of the evidence** | **Distal / Proximal  factor** | **Assessed  relevance** |
| --- | --- | --- | --- |
| ***Individual factors*** | | |  |
| **Age** | Most studies look at age, even just as a control variable. Despite a tendency to believe that there is a U-shaped relationship between age and loneliness, the evidence is mixed. In fact, the link between age and loneliness often ceases being statistically significant when other risk factors are taken into account in the analyses. | Distal | 0 |
| **Sex** | Men appear slightly lonelier than women if loneliness is measured indirectly, while it is often the other way around if direct measures are used. Just like for age, however, there is no clear evidence of a direct link between sex and loneliness. | Distal | 0 |
| **Race / ethnicity / migration**  **background** | Having a different ethnicity than the prevalent one in one’s country of residence tends to be associated with higher loneliness, but the effect goes probably through other channels (e.g. health, income or subjective feelings of belonging). | Distal | 0 |
| **Education** | No research has studied the relationship between loneliness and education specifically. The evidence from studies using education as a control variable is mixed. | Distal | 0 |
| **Employment** | Unemployment is linked to higher levels of loneliness but there is not strong evidence proving that this relationship is causal. In fact, there is possibly a bidirectional relationship between employment status and loneliness. | Distal | + |
| **Financial situation** | A worsened financial situation plausibly leads to increased feelings of loneliness, but possibly a part of the relationship is due to other related factors, such as health and social participation. | Distal | + |
| **Psychological factors** | It is mostly the Big Five personality traits that have been studied in terms of their link to loneliness. Extroversion and neuroticism (i.e., a long-term tendency to experience negative emotions, or its opposite, emotional stability) have been found to be linked to loneliness (negatively and positively, respectively). The evidence on agreeableness, openness and conscientiousness is less clear-cut. | Unclear | ++ |
| **Health** | The relationship between health and loneliness is bidirectional, i.e., ill health causes loneliness and loneliness causes worse health. Mental health (e.g. depression, anxiety) is often one of the strongest predictors of loneliness. There is some evidence that the link of physical health to loneliness is mediated through social activity or psychological factors. | Proximal | ++ |
| **Marital / partner status** | Partner status (or its change) is one of the most important direct determinants of loneliness (subject to a good relationship with this partner). Widows, people who are divorced or singles are among those most at risk of feeling lonely. | Proximal | +++ |
| **Living arrangements** | Living alone is strongly associated with more feelings of loneliness and living with others, especially a partner, is linked to less loneliness. In the case of old adults, living in nursing homes or residential care is possibly associated with higher levels of loneliness than community living. | Proximal | ++ |
| **Social network** | Frequent contact with friends and family, receiving and giving social and emotional support, and especially, good relationships with one’s social network, are major protective factors against feelings of loneliness. | Proximal | +++ |
| **Social activity** | There are less studies looking at social activity than on social networks, but the evidence suggests that being more socially engaged tends to be associated with lower levels of loneliness. Nevertheless, the relationship is possibly bidirectional and mediated by social network characteristics. | Proximal | + |
| ***Societal factors*** | | |  |
| **Living environment** | It is mostly the subjective evaluation of the neighbourhood and the access to different facilities or green spaces that matter for loneliness (probably through a better sense of belonging in the neighbourhood and easiness of socialising with other local inhabitants). | Distal | + |
| **Contextual factors** | There is, at the moment, insufficient evidence on the effects of culture or other country characteristics on loneliness. There is some evidence that individual- and country-level factors interact, so that the same situation may be perceived differently in different countries. | Distal | 0 |

*Note:* A distal factor is a factor that has an indirect influence on loneliness. A proximal factor has a direct influence on loneliness. Assessed relevance: 0 means limited relevance, + somewhat relevant, ++ relevant, +++ very relevant.

**Table A3**

*Summary of Individual Studies*

| **Study** | **Data** | **Risk factors of interest & Loneliness measure** | **Method(s)** | **Findings** |
| --- | --- | --- | --- | --- |
| Aartsen & Jylhä (2011) | • Finland • 1979-2006 • Tampere Longitudinal Study on Ageing • Longitudinal (4 waves) • N=469 at baseline (aged 60-89, 53.3% female, not lonely), N=463 at first follow-up, N=304 at second follow-up, N=31 at third follow up | Risk factors:  • Age • Social (partner status, having friends, being socially active) • Health (self-rated, physical disabilities) • Psychological (mood, nervousness, irritability, feelings of uselessness)  Loneliness measure: Single item: "Do you feel lonely?" (often/sometimes/never/don't know) --> binary, change at follow-up by 1 or 2 steps | Stepwise logistic regressions (bivariate and multivariate) | • In bivariate analyses, being a female and negative changes (not levels) in partner status, friends, social activity, physical health (not self-perceived health), low mood, nervousness, uselessness (but not irritation) are significantly associated to the onset of loneliness  • Sex, loss of friends and physical health are no longer significantly linked to the onset of loneliness in multivariate analyses, the other (change) variables still are (loss of a partner, reduction in social activity, increase in low mood, nervousness and uselessness) • Age is not linked to the onset of loneliness |
| Abdellaoui et al. (2019) | • Netherlands • 2004-2014 • Netherlands Twin Register • Longitudinal (3 waves) • N=29,625 (aged 18-98) • N=4,375 for genetic analysis • N=15,878 for twin analysis • N=4,436 spouse pairs | Risk factors:  • Demographic (age, sex) • Big Five personality traits • Genetic information  Loneliness measure: 3-item R-UCLA scale --> log-transformed score | • Generalised estimation equations • Genomic-relatedness-based restricted maximum-likelihood • Autoregressive cross-lagged panel model • Correlation analysis | • Only neuroticism is significantly associated with loneliness when controlling for other personality traits - they also show a strong genetic correlation • Twin analyses show that the difference in neuroticism between the two twins is highly correlated with the difference in loneliness and that differences in neuroticism and loneliness in time are strongly correlated • Longitudinal analysis shows a bidirectional positive relationship between loneliness and neuroticism (with the effect of neuroticism on loneliness being stronger than the other way around) • There is significant assortative mating for both loneliness and neuroticism, but not cross-trait assortative mating • Age is associated with higher loneliness |
| Altschul et al. (2021) | • UK • 2004-2013 • The Thirty-Six Day Sample (36DS) • The Lothanian Birth Cohort of 1936 (LBC1936) • Healthy Ageing in Scotland (HAGIS) • English Longitudinal Study of Ageing (ELSA) • Multiple cross-sections • N=792 (Mage = 77.5, 48% female) - "older cohort exploratory" analysis, 36DS • N=1,015 (Mage = 70, 49.3% female) - "older cohort confirmatory", LBC1936 • N=612 (aged 50-69, 56.2% female) - "younger cohort exploratory", HAGIS • N=6,106 (Mage = 59.3, 55.9% female) - "younger cohort confirmatory", ELSA | Risk factors:  • Sex • Socio-economic (social class, education) • Health (general cognitive function, subjective) • Social (number of children, marital status, living arrangements) • Personality  Loneliness measure:  • Single question (older cohorts): "At the present moment, do you feel lonely?" (never/seldom/only occasionally/quite often/most of the time) --> score  • 6-item de Jong Gierveld scale (younger cohort exploratory) --> 1-3 mean score • 3-item modified UCLA scale (younger cohort confirmatory) --> 1-3 mean score | • Extreme gradient boosting modelling • Ordinal regression models | • In the older cohorts, emotional stability and subjective health have a negative relationship with loneliness • Living alone has a positive relationship and interacts with sex and emotional stability - men living alone are more likely to be lonely than women, but women not living alone more likely to be lonely than men, while less emotionally stable people living alone are lonelier than more emotionally stable people living alone • Being widowed has a positive effect on loneliness (but only in exploratory analysis) • Social class and sex have no effect on loneliness • Of the significant predictors of loneliness in the older cohorts, only emotional stability and extroversion are significantly (negatively) related to loneliness also in the younger cohorts |
| Arpino et al. (2022) | • Multiple European countries and Israel • 2019-2020 • Survey of Health, Ageing and Retirement in Europe (SHARE)  • SHARE Corona Survey 1 (SCS1) • Longitudinal (2 waves) • N=44,329 – SHARE  • N=53,820 (aged 50+, 57.8% female) – SCS1 | Risk factors:  • Demographic (age, sex, country of residence)  • Socio-economic (education, employment status) • Social (partnership status, being a parent)  • Health (self-reported health conditions, activity limitations)    Loneliness measure:  • Single question: “How much of the time do you feel lonely?” (often/some of the time/hardly ever/never) --> binary (top 2 categories collapsed)  • Single question (only in SCS1 and for those answering in top 2 categories to the first single question): “Has that been more so, less so, or about the same as before the outbreak of Corona?” --> binary (more and about the same collapsed) | • Logistic regression  • Multinomial logistic regression | • Before COVID-19, unpartnered parents or partnered childless were at highest risk of loneliness  • During COVID-19, unpartnered and childless remained at higher risk of loneliness  • The unpartnered were more likely to start feeling lonely during the pandemic, and both childless and unpartnered were more likely to remain lonely during the pandemic if they were lonely already before  • Being a female, older, with lower education and having bad health were associated with loneliness before and during the pandemic, while having a job (compared to being retired) was linked to less loneliness  • Being a female, of higher age and having bad health were also associated with feeling lonelier during the pandemic than before, while having a job (compared to being retired) was linked to feeling less lonely than before |
| Barreto et al. (2021) | • 237 countries, islands and territories • 2018 • BBC Loneliness Experiment • Cross-section • N=46,054 (aged 16-99, 67.7% female) | Risk factors:  • Demographic (age, sex) • Individualism of the country  Loneliness measure: UCLA 4-item scale --> 1-4 mean score | Hierarchical regressions | • Age is negatively associated with loneliness, while living in an individualistic country is positively associated with loneliness  • Decrease in loneliness with age is stronger in collectivist countries and for males  • Men are lonelier than women at all ages, but the difference is smaller in oldest groups and bigger in individualistic nations  • People in individualistic countries are always lonelier than those in collectivist countries, but the difference is bigger for older participants and for males |
| Bayat et al. (2021) | • Netherlands • 2015 • National Youth Health Monitor • Cross-section • N=7,956 (Mage = 14.3, 50.4% female) | Risk factors:  • Demographic (age, sex, ethnicity) • Contextual (family - parental divorce and illness of a family member, school - bullying victimisation and student-teacher relationship, social media - cyberbullying and problematic social media use)  Loneliness measure: Single item: "Do you ever feel lonely?" (yes, often/yes, sometimes/seldom/no) --> binary (top 2 categories collapsed) | Univariate and multivariate logistic regressions | • All variables are positively related to the likelihood of being lonely (age, female sex, non-Dutch ethnicity, parental divorce, sick family member, being bullied in school and online, poor student-teacher relationship and problematic social media use) in univariate analysis • In multivariate analysis, the effects are confirmed though they weaken a bit (except those of demographic characteristics) and cyberbullying stops being significant |
| Beutel et al. (2017) | • Germany • 2007-2012 • Gutenberg Health Study • Cross-section • N=15,010 (aged 35-74, 49.4% female) | Risk factors:  • Demographic (age, sex) • Socio-economic (status, unemployment) • Social (having a partner, having children, living alone) • Physical health (smoking, BMI, alcohol consumption, antidepressant and anxiolytic consumption, doctor visits, inpatient treatment) • Mental health (depression, anxiety, panic attacks, suicidality, distressed personality)  Loneliness measure: Single item: “I am frequently alone/have few contacts” (no, does not apply/yes it applies, but I do not suffer from it/yes, it applies, and I suffer slightly/yes, it applies, and I suffer moderately/yes, it applies, and I suffer strongly) --> categories (no/slight/moderate/severe loneliness) | • Kruskal-Wallis • Chi-squared tests | • Younger participants are lonelier than the older ones • Loneliness is less prevalent among men, people with children or a partner, and decreases with increasing alcohol intake • Loneliness is more prevalent among those living alone, with a lower socio-economic status, for smokers, increases with antidepressants and anxiolytics intake, healthcare utilisation (both physician and inpatient) and all measures of psychological distress • Employment and BMI do not impact loneliness • Sex interacts with living arrangements and age - loneliness is higher for women living with a partner at all ages, but for those living alone, women are lonelier than men only in 35-44 and 55-64 age categories; peak of loneliness for women is at age 35-44, while for men at 45-54 |
| Böger & Huxhold (2018) | • Germany • 1996-2011 • German Ageing Survey • Longitudinal (4 waves) & cross-section • N=11,010 (aged 40-84, 49.1% female) | Risk factors:  • Social (network size, social activity - score based on time spent participating in different activities, self-perceived relationship quality with friends and family) • Physical health • Negative affect  Loneliness measure: 6-item de Jong Gierveld scale --> overall score | Bivariate and multivariate autoregressive models | • Smaller network size leads to more loneliness, but also more loneliness leads to smaller network size - the latter effect is stronger and both effects get stronger with age • The very same holds for interrelations between social activity and loneliness, and negative affect and loneliness (except that both directions of the relationship between negative affect and loneliness get weaker with age) • Loneliness doesn't lead to increased health problems if negative affect is controlled for (it mediates the effect), but health problems do have a strong impact on loneliness (also through increased negative affect) |
| Bonsaken et al. (2021) | • Norway, US, UK, Australia • 2020 • Survey about COVID-19 • Cross-section • N=3,474 (aged 18+, 73.3% female) | Risk factors:  • Demographic (age, sex) • Socio-economic (education, employment status) • Cohabitation (with partner/spouse or not) • Pandemic-related (concerns about health/family/future/finance, risk assessment for oneself and local area, protective measures - quarantine, self-isolation) • Social media use  Loneliness measure: 6-item de Jong Gierveld scale --> 0-24 score for overall loneliness and 0-12 scores for social and emotional loneliness | • ANOVA • t-tests • Correlation analysis • Linear regressions | • Higher age is linked to more social loneliness, but less emotional loneliness • Females experience more emotional loneliness and less social loneliness • Higher education is negatively linked to social and overall loneliness, while living with spouse/partner protects against all types of loneliness • Concerns about health increase emotional loneliness, concerns about future and finances increase all types of loneliness (and are the strongest predictors) and more time spent on social media is linked to higher emotional and overall loneliness • Employment, concerns about next of kin, risk perceptions and quarantine/self-isolation are not linked to loneliness |
| Bosma et al. (2015) | • Netherlands • 2012 • Epidemiologic monitoring survey • Cross-section • N=10,029 (aged 17-65, with at least 1 of 19 illnesses listed in the past 12 months) | Risk factors:  • Demographic (age, sex, ethnic background) • Socio-economic (net income, education, employment) • Marital status  Loneliness measure: 11-item de Jong Gierveld scale --> binary (score 9 or more) | • Chi-square tests • Logistic regressions | • People at the bottom income quintile are more likely to be lonely and have less social contacts compared to those in the top income quintile • Being divorced and work disabled partially explain the link between income and loneliness and social contacts |
| Bu et al. (2020) | • UK • 2017-2020 • Understanding Society: the UK Household Longitudinal Study • UCL COVID-19 Social Study • Multiple cross-sections • N=31,064 (aged 18+, 51.8% female) for 2017-2019 analysis  • N=60,341 (aged 18+, 49.8% female) for 2020 analysis | Risk factors:  • Demographic (age, sex, ethnicity) • Socio-economic (employment, education, income) • Living status • Area of living (urban/rural)  Loneliness measure: • 3-item UCLA scale --> 3-9 score • Single item on frequency of feeling lonely (hardly ever or never/some of the time/often) | OLS regressions | • Loneliness is higher during the COVID-19 pandemic, but the risk factors are nearly identical to before the pandemic • Age and income are negatively associated with loneliness both before and during the pandemic, while living alone is positively associated with loneliness both before and during, and all these effects get stronger during the pandemic • Being unemployed, inactive or a student are associated with loneliness both before and during the pandemic, but the student status more strongly during the pandemic • Being a woman, of non-white ethnicity, with lower education and living in an urban area are all positively associated with loneliness before and during the pandemic, but the effects are small |
| Bu et al. (2020b) | • UK • 2020 • UCL COVID-19 Social Study • Longitudinal (7 waves) • N=38,217 (aged 18+) | Risk factors:  • Demographic (age, sex, ethnicity) • Socio-economic (employment, education, income) • Social (living status, friend network, usual social contact frequency, perceived social support) • Clinical diagnoses of depression, anxiety or other psychiatric conditions • Area of living (urban/rural)  Loneliness measure: 3-item UCLA scale --> 3-9 score | Growth mixture modelling | • Age, living with others, living in a rural area, having more close friends and receiving higher social support are all negatively linked to loneliness • Being a woman, having a low income and a diagnosed mental health condition are all associated with increased loneliness • Education, employment and ethnicity do not have a significant link to loneliness, but there is some positive effect of being a student or inactive and of frequency of usual face-to-face contact |
| Buecker et al. (2021) | • Netherlands • 2008-2017 • Dutch Longitudinal Internet Studies for Social Sciences • Longitudinal (up to 10 waves) • N=13,945 nested in N=8,685 households (54.3% female), propensity score-matched | Risk factors:  • Demographic (age, sex) • Socio-economic (education, monthly income) • Family-related major life-events (widowhood, divorce, marital separation, marriage, cohabitation, transition into parenthood) • Work-related major life-events (transition into paid employment, job loss, retirement)  Loneliness measure: 6-item de Jong Gierveld scale --> overall score | • Propensity score matching • Generalised additive models • Linear mixed effects models | • Cohabitation - there was no anticipatory reaction or no post-event change in loneliness, but a group difference in loneliness between the cohabiting group and their propensity score-matched counterparts • Marriage - those who got married were on average less lonely than those who didn't and there were changes in loneliness before and after the event only for those who got married younger or older than at the average (normative) age • Child - those having the first child during the survey were on average less lonely than those who didn't, there was no anticipatory effect, but a significant decrease in loneliness in the year after the birth of the child and then a linear increase, but no long-lasting change • Marital separation - those who separated during the study reported higher levels of loneliness than those who never did, loneliness linearly increased before the event and linearly decreased after • Divorce - those who divorced reported higher levels of loneliness compared to those who did not, and there was an increase in loneliness before the event but no reaction to it • Widowhood - there were no group differences in loneliness, no linear anticipatory changes, but a post-event reaction (higher loneliness scores after the event compared to before, with signs of adaptation) • Transition into paid employment - there were no group differences in loneliness, no anticipatory reaction, but an increase in the years after the event  • Job loss - those who have experienced job loss reported higher loneliness than those who did not, and loneliness linearly increased before the event and then decreased more slowly after (+there was an extra effect in the first year after the event) • Retirement - there were no differences between groups, no anticipatory or first-year reaction to the event, but overall a decrease in loneliness |
| Buecker et al. (2021b) | • Germany • 2013 • German Socio-Economic Panel Study • INKAR database • Cross-section • N=17,602 (aged 18-103, 54% female) | Risk factors:  • Demographic (age, age^2^, sex, migration status) • Socio-economic (education, employment, income) • Social (relationship status, frequency of contact with family and friends) • Subjective health • Self-reported neighbourhood characteristics (walking distance to public transport, parks, leisure facilities, distance to city centre, perceived neighbour relationships) • Objective regional and neighbourhood characteristics (regional level mean age, socioeconomic deprivation, population change, population density, distance to nearest regional centre) • Residential region (East/West Germany) • Urban/rural area  Loneliness measure: UCLA 3-item scale (adapted) --> overall score | • Actor-based clustering approach • Sequential linear mixed-effects models | • From individual-level predictors, being a female, being single, being a migrant and having a longer distance to reach parks and leisure facilities have a positive link to loneliness, while being older, having a higher income, being employed, having a more frequent contact with family and friends, being in better health and having better relations with neighbours have a negative relationship with loneliness • Education, not living with a partner (compared to living with them), distance to public transport, the nearest city centre, and living in a rural area do not impact loneliness • Living in East Germany is linked to higher loneliness when other individual factors are controlled for, but when regional-level factors are added, the effect disappears • From the regional-level predictors, higher population growth and higher remoteness from the regional centres are linked to more loneliness, while age composition, socio-economic deprivation and population density do not impact loneliness |
| Caro et al. (2022) | • Germany, France, Italy, Spain, Sweden • 2020-2021 • COME-HERE Panel Survey • Longitudinal (5 waves) • N=3,172 (aged 18+, 51% female) | Risk factors:  • Demographic (age, sex) • Socio-economic (education, income) • Social (living with a partner, change in frequency of remote and face-to-face interactions) • Health (mental health condition, physical medical condition, physical activity) • Pandemic-related (lockdown stringency, daily COVID-19 cases and deaths, isolation, absence of any negative experiences)    Loneliness measure: UCLA loneliness scale-8 --> 8-32 score | • Latent class modelling | • Higher age, higher levels of education, living with a partner, and physical activity are linked to lower levels of loneliness • Decrease in social interactions, particularly the face-to-face ones, having any health conditions or having to isolate due to COVID-19 are associated with higher levels of loneliness  • Lockdown stringency has a positive impact (i.e., increases) loneliness for those with lower levels of loneliness • Income, and COVID-19 cases or deaths are not statistically significantly linked to loneliness |
| Choi et al. (2022) | • US  • 2020  • Understanding America Study COVID-19 Survey  • Cross-section  • N=3,253 (aged 50+, 46% female) | Risk factors:  • Demographic (age, sex, race/ethnicity)  • Socio-economic (education, income)  • Household composition  • Pandemic-related (official social distancing policies, social distancing measures in terms of avoiding public spaces/gatherings/crowds, cancelling/postponing personal/social activities, social visits, no close contact with people living together or people not living together)    Loneliness measure: Single-item question: “In the past 7 days, how often have you felt lonely?” (Not at all or less than 1 day/1-2 days/3-4 days/5-7 days) --> binary (top 3 categories collapsed) | • Multivariate logistic regression models | • Lower age, being a female, living alone or with other people but not a partner (compared to living with a partner) or having very low income linked to higher levels of loneliness  • Being of Black or Hispanic race (but not Asian or other race) is linked to lower levels of loneliness compared to being of White race  • Education, governmental social distancing policies, avoiding gatherings or public places, and social visits and close contacts with people not living together are not linked to feelings of loneliness  • Cancelling or postponing social activities and avoiding close contact with people living together are associated with greater odds of loneliness  • Limiting close contact with co-residents increases the probability of loneliness more for males, non-Hispanic Whites, and those with higher levels of education and income |
| Dahlberg et al. (2018) | • Sweden • 1992-2014 • Swedish Panel Study of Living Conditions of the Oldest Old • Repeated cross-sections (5) • N=2,572 (aged 77+) for analysis of trends (5 time-points) • N=1,962 (aged 70+) for analysis of factors (2 time-points) | Risk factors:  • Demographic (age, sex) • Education • Social (marital status, social support, social contacts) • Limitations in activities of daily living (ADLs) • Psychological distress  Loneliness measure: Single item: "Are you ever bothered by feelings of loneliness?" (almost never/seldom/often/almost always) --> categories or binary (top 2 categories collapsed) | • Chi-square tests • Linear regressions • Logistic regressions | • Women are lonelier than men (significant in 3/5 waves), but the effect of sex disappears if other factors are controlled for • Not being married or being widowed are linked to a higher likelihood of loneliness (the former has a significant effect only in 2004, not in 2014), and so are lacking access to social support, having fewer social contacts, reporting ADL limitations (but the effect is significant only in 2004) and suffering from psychological distress (severe problems are the strongest predictor of loneliness) • Age and education level have no significant effect on the likelihood of feeling lonely when other factors are controlled for |
| Dahlberg et al. (2022) | • Denmark, Finland, Norway, Sweden  • 2006-2014  • European Social Survey  • Repeated cross-sections (4)  • N= 7,755 (aged 60+, 48-53.1% female) | Risk factors:  • Demographic (age, sex) • Socio-economic (education, exclusion from material resources – household income, income concern)  • Social (exclusion from social relations – social contacts, emotional support and household size, civic exclusion – political participation, voting behaviour)  • Neighbourhood exclusion  • Self-rated health  Loneliness measure: Single item: “How much of the time during the past week have you felt lonely?” (none or almost none of the time/some of the time/most of the time/all or almost all the time) --> binary (top 3 categories collapsed) | • Hierarchical logistic regression | • Higher age is associated with more loneliness in Finland and Sweden, being a woman in Norway and Sweden, health limitations only in Finland and self-rated health in all countries except Finland, while education is not linked to loneliness  • Lower frequency of social contacts and living alone (compared to a two-person household) are associated with a higher probability of loneliness in all countries, with the latter being the strongest predictor of loneliness  • Lower neighbourhood safety associated with a higher probability of loneliness in Sweden and Denmark, income concern in Sweden and Finland, and no emotional support in all countries except Norway  • Civic participation and household income are not associated with feelings of loneliness |
| Das (2021) | • 10 EU countries • 2011-2017 • Survey of Health, Ageing and Retirement in Europe (SHARE) • Longitudinal (4 waves) • N= 37,695 (aged 50+, 55% women) | Risk factors:  • Age • Socio-economic (education, household wealth)  • Social (family ties – frequency of interactions with children and parents, household size, social activities)  • Health (chronic conditions, functional limitations, cognitive capacity, poor vision and hearing)  • Country-level secularism  Loneliness measure: 3-item UCLA scale --> mean score | • Multilevel linear latent growth models | • Societal secularism is not associated with loneliness  • Family ties are associated with lower loneliness (only at baseline, not associated to trends in loneliness) |
| de Jong Gierveld et al. (2009) | • Netherlands • 2001-2002 • Dutch Living Arrangements and Social Networks of Older Adults Survey • Longitudinal Ageing Study Amsterdam • Cross-section • N=755 (aged 64-92, 39.6% female, living with a spouse) | Risk factors:  • Demographic (age, sex) • Income • Social (children - number and contact, religious activities, social network size, giving and receiving instrumental and emotional support) • Partner-related (partner history, cognitive functioning of the spouse, marital functioning and quality - frequency of important conversations, degree of spousal agreement, spouse as confidant, quality of sex life) • Functional limitations in daily activities  Loneliness measure: 11-item de Jong Gierveld scale --> overall, social and emotional scores | Hierarchical negative binomial regression analyses | • Men's social loneliness is significantly higher than women's, but there is no sex difference in emotional loneliness • Age, income, religious affiliation and functional limitations are linked to higher emotional loneliness only if other factors are not controlled for, and income and religious affiliation are linked to higher social loneliness only if other factors are not controlled for • Emotional loneliness is linked to having children but not seeing them on a weekly basis, smaller network size, and spouse's functional limitations • (Re-)marriage protects from emotional loneliness (more so for men than women), and so do giving instrumental support (to spouse and others), receiving emotional support from spouse and good sex life (other indicators of marriage quality do not have an impact) • Emotional and instrumental support received not from spouse and spouse's cognitive problems are unrelated to emotional loneliness • Social loneliness is linked to having children and seeing them frequently, network size, emotional support received (from spouse and others), functional limitations of spouse (this is important only for men), instrumental support given to others (not spouse), frequency of good conversations with the spouse and good sex life (other indicators of marriage quality are unrelated to social loneliness) • Age, functional limitations, instrumental support and cognitive problems of the spouse are unrelated to social loneliness |
| de Jong Gierveld et al. (2012) | • 5 European countries • 2004-2011 • Generations and Gender Surveys • Cross-section • N=12,756 (aged 60+, 50-69% female depending on the country) | Risk factors:  • Demographic (age, sex) • Perceived financial situation • Living arrangements • Family-related (intergenerational support received and given, having children alive) • Health  Loneliness measure: 6-item de Jong Gierveld scale --> 0-6 score | • Latent class analyses • ANOVA • Regression analyses | • Older people are lonelier in Eastern than Western Europe • Older people living alone are the loneliest, followed by those living with children and finally those living with a partner • Older people who are mainly receiving intergenerational support are the loneliest, followed by those who both give and receive it and those with a low likelihood of exchanges and get togethers, and the least lonely are the ones that are mostly giving intergenerational support • In both Eastern and Western Europe, high likelihood of giving support and moderate to high likelihood of get togethers protect from loneliness (especially if both are high) - this holds only weakly for those coresiding with their children and partner in Eastern Europe, and not at all for those coresiding with their children without the partner in Eastern Europe • In Eastern Europe, receiving support and coresiding with children without the partner is linked to higher loneliness, there is no effect of receiving support if coresiding also with a partner, and if living alone, receiving support is linked to lower loneliness |
| de Jong Gierveld et al. (2015) | • Canada • 2008 • Statistics Canada's General Social Survey • Cross-section • N=3,692 (aged 65+, 52-58% female depending on ethnicity) | Risk factors:  • Demographic (age, sex, migrant status) • Socio-economic  • Social - micro-level (marital status, frequency of contact with friends and family, satisfaction with this frequency, number of close relatives and friends) • Social - meso-level (participation in community organisations, informal involvement in neighbourhood, sense of belonging in the local community) • Social - macro-level (mother tongue of contacted friends, number of ethnically different contacted friends) Self-reported health  Loneliness measure: 6-item de Jong Gierveld scale --> overall score | • Multivariate hierarchical regressions  • UNIANOVA models | • Descriptively, loneliness is significantly lower for Canadians than for immigrants from non-European countries (these have the highest loneliness scores) and of European but non-French and British origin, and there is no difference in loneliness between Canadians and immigrants of French or British origin • In the multivariate models, only the non-European immigrants are lonelier than Canadians • Number of close relatives and friends, and satisfaction with the contact with these are strongly associated with loneliness, while frequency of contact with friends, not relatives, is associated with loneliness • Sense of belonging to local community is strongly and significantly linked to loneliness, while participation in community groups and helping neighbours have no impact • Having most friends with the same mother tongue has a significant effect on loneliness, while sense of belonging in Canada and having ethnically different friends have no effect • Being divorced/widowed/never married and being in poor health are associated with higher loneliness, while sex has no effect |
| Ejlskov et al. (2017) | • UK • 2014-2015 • National Survey of Health and Development • Cross-section • N=2,453 (aged 68, 52% female) | Risk factors:  • Demographic (age at retirement, sex) • Socio-economic (occupational social class, home ownership, number of cars in the household, education, retirement) • Social (marital status and change, frequency of meeting friends/relatives, number of friends/relatives seen frequently, voluntary work, number of children and grandchildren, living close to children, frequency of visits by grandchildren, death of children, frequency of participation in social activities, in church, frequency of prayer and meditation, seeing purpose in religion, importance of faith, closest person, emotional support and negative aspects of the relationships) • Health (self-rated, longstanding illness/problem and degree to which this limits daily activities) • Psychological (personality characteristics - extroversion/agreeableness/neuroticism/conscientiousness/personal mastery, affective states - mental wellbeing/fatigue from participating in or hosting a social activity)  Loneliness measure: UCLA 3-item scale --> 3-9 score | Recursive partitioning (random forest) | • Wellbeing, personal mastery, extroversion, number of visits to friends/relatives, number of friends/relatives seen in the past month, low degree of limiting health problems, good self-rated health, being married and identifying the spouse as the closest confidant are the most important correlates of loneliness • The least important correlates of loneliness are neuroticism, getting divorced or widowed in the past 6 years, seeing religious faith as important and providing meaning, having a longstanding health issue, civic participation (political, recreational, community service) and number of cars owned |
| Entringer & Gosling (2022) | • Germany • 2020 • SOEP-CoV project • Longitudinal (2 waves) • N=6,010 (aged 18+, 60.9% female) | Risk factors:  • Demographic (age, sex, migration background)  • Socio-economic (income, employment status)  • Household size  • Big Five personality traits  • Pandemic-related (work from home, decrease in income)    Loneliness measure: 3-item UCLA Loneliness Scale (adapted) --> overall score | • Local structural equation modelling | • Increase in loneliness during the pandemic was experienced mostly by women (sex being the strongest predictor), extroverted, neurotic, and conscientious individuals, as well as those whose income decreased during the pandemic  • Higher age and income, and being unemployed before the pandemic were associated with lower increases in loneliness during the pandemic  • Migration background, household composition and working from home were not associated with increase in loneliness during the pandemic |
| Fokkema et al. (2012) | • 14 European countries • 2006-2007 • Survey of Health and Retirement in Europe • Cross-section • N=12,248 (aged 50-104, 52.5-59.3% female depending on the country, non-institutionalised) | Risk factors:  • Demographic (age, sex) • Socio-economic (education, employment status, perceived difficulties with current income) • Social (marital status, having children and frequency of contact with them, having living parents and frequency of contact with them and their health, social participation, help with personal care given, informal help given and received, taking care of grandchildren) • Health (perceived, problems with seeing or hearing, functional limitations)  Loneliness measure: Single item about whether or not they felt lonely much of the time during the past week (yes/no) --> binary | Multivariate logistic regressions | • Higher levels of loneliness are found in Southern and Eastern Europe, lower in Western and Northern Europe • Age, having a paid job, having children (and having more frequent contact with them), social participation, giving help to family members, and having grandchildren and looking after them more frequently are linked to a lower likelihood of being lonely • Being a female, being divorced, widowed or never married, having more income difficulties, worse self-rated health, more instrumental functional limitations, poorer seeing, giving help with personal care to a household member and receiving informal help from family are linked to a higher likelihood of loneliness, with marital status being the strongest predictor • Education, functional limitationss, bad hearing, having living parents and frequency of contact with them, and giving or receiving help not to and from family members do not have an impact on the likelihood of feeling lonely • Demographic composition explains higher levels of loneliness in the Czech Republic and Greece, limited socio-economic resources and poor health explain higher levels of loneliness in Spain, Italy, Czech Republic and Poland, while higher loneliness in Italy and France are less well explained by these predictors, and social network characteristics do not contribute much to explain cross-country differences in loneliness (even though they are an important predictor of loneliness) |
| Fokkema & Naderi (2013) | • Germany • 2005-2006 • German Generations and Gender Survey • Cross-section • N=3,742 (aged 50-79, non-institutionalised, born in Germany or Turkey) | Risk factors:  • Demographic (age, sex, ethnicity) • Socio-economic (education, employment, perceived financial situation, financial support) • Social (partner and quality of relationship, children - co-residence and contact and relationship quality, emotional support, personal care given and received) • Health (subjective, objective - based on chronical illness and disability) • Satisfaction with dwelling  Loneliness measure: 6-item de Jong Gierveld scale --> 0-6 score | Multivariate analysis | • Turks are lonelier than natives, but when all other factors are controlled for, the effect disappears • Women are less lonely than men • Age, being unemployed, being chronically ill or having physical limitations and lack of providing emotional support all have a significant positive relationship with loneliness, but this vanishes when other factors are controlled for • Better education, better perceived financial situation, better perceived health, receiving emotional support, looking after grandchildren and higher satisfaction with dwelling are all inversely associated with loneliness • Living with a partner protects from loneliness, but only if the relationship is good • Living with a child or having a good relationship (more than frequent contact) with them also protects from loneliness, though the effect of quality if the contact frequency is low disappears in the full model • Financial support and personal care are not linked to loneliness |
| Franssen et al. (2020) | • Netherlands • 2016 • Adult Health Monitor Limburg 2016 • Cross-section • N=26,319 (aged 19-65, 49-50% female depending on age group) | Risk factors:  • Demographic (age, sex, ethnicity) • Social (marital status, living arrangements, volunteer work, frequency of social contact with family/friends/neighbours, social exclusion, informal caregiving activity) • Socio-economic (education, financial imbalance, employment status) • Health (general, limitation in daily activities, chronic illness) • Psychological (depression or anxiety disorder, psychological/social/emotional wellbeing)  Loneliness measure: 11-item de Jong Gierveld scale --> binary (score 3 or more) | Bivariate and multivariate logistic regressions | • Loneliness increases with age • In bivariate analyses, all included factors are significantly associated with the likelihood of being lonely in all age categories • Living alone, lower frequency of contact with neighbours, social exclusion, psychological distress, and lower emotional and psychological well-being are all associated with a higher likelihood of feeling lonely in multivariate analysis in all age groups with similar magnitudes • Non-Dutch ethnicity, perceived financial imbalance and non-frequent contact with friends are linked to the likelihood of reporting loneliness in all age groups, but with varying degrees of magnitude • Sex is not important for the likelihood of loneliness in young adults (men are more likely to be lonely in other age groups), while education is only important for these (higher education is linked to a lower likelihood of being lonely) • Employment status matters for the likelihood of loneliness only for early middle-aged adults (being employed is linked to a lower likelihood of loneliness), while not being married and worse self-reported general health are associated with a higher likelihood of being lonely only for older middle-aged adults, and less contact with family is linked to a higher likelihood of loneliness for both these groups • Volunteer work, limitations in daily activities, having a chronic disease and being a caregiver are not associated with the likelihood of being lonely |
| Greenfield & Russell (2011) | • US • 2005-2006 • National Social Life, Health, and Aging Project • Cross-section • N=2,888 (aged 57-85, 51% women) | Risk factors:  • Demographic (age, sex, race) • Socio-economic (education, employment) • Living arrangements (with spouse or partner/with spouse or partner and children/with spouse or partner and other relatives or friends/singles with children/singles with other relatives or friends/singles alone) • Functional limitations  Loneliness measure: 3-item Revised UCLA scale and single item about frequency of feeling lonely over the past week (4 options) --> standardised mean score (merging the two measures) | OLS regressions | • Compared to living with only a partner, those single (living alone/with children/with others) are lonelier, while there is no difference for those living with a partner and children, or partner and others • Associations between living alone and loneliness, and being single and living with others and loneliness are stronger for men than women, while that between being single and living with children and loneliness is stronger for women • Sex, employment and race do not have an impact on loneliness • Age has a very small and negative effect, education has a negative effect, and functional limitations have a positive and strong effect on loneliness (but not as much as living arrangements) |
| Groarke et al. (2020) | • UK • 2020 • COVID-19 Psychological Wellbeing Study • Cross-section • N=1,989 (aged 18-87, 70.4% females) | Risk factors:  • Demographic (age, sex) • Socio-economic (self-rated income level, employment status, education) • Social (relationship status, number of adults and children in the household, social support) • Health (physical or mental health conditions, sleep quality, PTSD,  • Psychological (anxiety and depression, emotional dysregulation) • Area • Country of residence (within UK) • COVID-19-related (current living situation, caring for someone with COVID-19, being employed as a key worker, current or past quarantine)  Loneliness measure: 3-item UCLA scale --> binary (score 6 or more) | • t-tests • Chi-square tests • Univariate and multivariate logistic regressions | • In multivariate analyses, age, social support, household size and being married (compared to being single) are linked to a lower likelihood of feeling lonely • Being separated or divorced (compared to being single), depressed, having worse sleep quality during COVID-19 and emotional regulation difficulties are linked to a higher likelihood of loneliness • Sex, country of residence, employment, income, education, COVID-19-specific variables, urbanicity and physical health conditions are not related to the likelihood of reporting loneliness • Age is the strongest predictor of the likelihood of being lonely |
| Guthmuller (2022) | • 27 European countries and Israel • 2015 • Survey of Health, Ageing and Retirement in Europe (SHARE) and SHARELIFE • Cross-section • N=27,623 (aged 50+, 57.1% female) | Risk factors:  • Demographic (age, sex)  • Socio-economic (subjective wealth in childhood, education, employment status, wealth)  • Personality traits  • Social (childhood circumstances – having friends, relationship with parents, never being physically harmed and religion, adult circumstances – marital status, household size, social network, size of the network, frequency of contact, mean closeness and geographical proximity of the network, social participation, computer skills)  • Health (subjective health in childhood, chronic diseases, depression, activities limitations)    Loneliness measure: 3-item Revised UCLA scale --> binary (being in the country-specific fourth quartile of the overall score) | • Stepwise multivariate logit model | • Ill health is the main factor associated with loneliness, followed by social support, personality traits, life circumstances during childhood, demographic and socio-economic factors and country-level characteristics  • Having friends, good relationship with mother, being physically harmed and not being religious, all during childhood, are related to loneliness in later life, while relationship with the father, being an only child, being in ill health and having low wealth in childhood are not linked to loneliness later in life (at least after controlling for other variables related to adult life)  • Extraversion, higher age, being employed (compared to being retired), being married, living with others, and having a better social network (bigger, closer, meeting more frequently) are associated with lower levels of loneliness  • Neuroticism, low education, participating in few social activities and being unhappy about it, having bad computer skills or having physical or mental health problems are associated with higher levels of loneliness  • Agreeableness, openness, conscientiousness, income, female gender and area of living are not linked to feelings of loneliness (at least when other variables are taken into account) |
| Hajek & König (2020) | • Multiple European countries (+Israel) • 2013-2017 • Survey of Health, Ageing and Retirement in Europe (SHARE) • Longitudinal (3 waves) • N=101,909 observations (aged 50+, 56.8% female, non-institutionalised, with a change in loneliness between waves) | Risk factors:  • Age • Income • Marital status • Health (self-rated, functional decline, cognitive functioning, chronic illnesses count score) • Depressive symptoms  Loneliness measure: 3-item Revised UCLA scale --> 3-9 score | Linear fixed effects regression | • Positive change (increase) in loneliness is associated with increasing age (small), worsening self-rated health (small), functional decline and increased depressive symptoms • Negative change (decrease) in loneliness is associated with being married, higher income (small) and better cognitive functioning (small) • There is no effect of changes in number of chronic illnesses on loneliness |
| Hansen & Slagsvold (2016) | • 11 European countries • 2004-2011 • Generations and Gender Survey • Cross-section • N=132,319 (aged 18-80) for loneliness and age analysis • N=33,832 (aged 60-80) for analysis with multiple factors | Risk factors:  • Age • Social (living arrangements, number of children) • Health (subjective, disability) • Socio-economic (educational level, being employed, financial situation - perceived difficulties in making ends meet)  Loneliness measure: 6-item de Jong Gierveld scale --> 0-12 score | • Chi-square tests • F-tests • OLS regressions • Ordinal probit model | • Loneliness increases with age, more so for women than men, more so in Eastern than North-Western European countries • In the 60-80 age group, living with a partner protects from loneliness (more for men), so does higher education level, better financial situation, higher number of children (more for women), and better health (more for women)  • Disability is linked to higher loneliness only among women aged 60-80  • Being employed does not impact loneliness in the 60-80 age group |
| Hawkley et al. (2020b) | • US • 2014-2018 • General Social Survey • Repeated cross-sections (2) • N=2,440 (aged 18-89, 53.9% female) | Risk factors:  • Demographic (age, sex, racial/ethnic minority status) • Socio-economic (income, education, work status) • Social (household size, children in household, legal marital status, frequency of taking part in religious activities other than services, frequency of spending an evening with family, friends, neighbours) • Perceived health  Loneliness measure: UCLA 3-item scale --> standardised mean score | • Locally weighted scatterplot smoothing • Regression analyses | • Loneliness has a non-linear relationship with age - it is the highest for young and oldest old adults, with another peak at around 50-60 years, and with two dips at around 40 and 70 years • Loneliness is higher for those not married (especially widowed), with a lower income, living alone (only as opposed to those living in 2-member household, not so much 3-member household), with poorer health and spending less evenings with neighbours and family, while age, sex, ethnicity, education, work status, living with children, frequency of contact with friends and religious engagement do not impact loneliness • Household size weakly interacts with age - the protective effect of 2-member household against loneliness is attenuated for those much younger or older than average, while other factors do not interact with age • Deviating from age-norm on income, health, marital and work status significantly affects loneliness (positively for income and health and negatively for marital and work status), but when regressed together with absolute levels, these effects become insignificant, while there is no effect of non-normative level of education, household size and children in the household, religious engagement or frequency of social interactions |
| Hawkley et al. (2008) | • US • 2002 • Chicago Health, Aging and Social Relations Study • Cross-section • N=229 (aged 50-65, 52.4% female) | Risk factors:  • Demographic (age, sex, race/ethnicity) • Socio-economic (education, household income, employment status) • Social (marital status, regular church attendance, group membership, social network size and frequency of contact, spousal confidant, overall network satisfaction) • Health (chronic conditions, number of symptoms, restrictions in activities of daily living) • Chronic stress exposure • Life event count  Loneliness measure: Revised 20-item UCLA scale --> 20-80 score | OLS regressions | • Age and sex are not related to loneliness (though in the full model, women are significantly less lonely than men) • Those of Hispanic (and marginally Black) ethnicity are lonelier than those with White ethnicity - these differences are explained by education and income • Having a high school diploma and a higher income is associated with lower loneliness, and the effect of income is partially explained by health variables • From health variables, only the number of symptoms is significantly (positively) related to loneliness • Being a group member is linked to significantly lower loneliness, while being married, retired or unemployed and attending church regularly do not have an effect on loneliness • The effects of having a high school diploma and health symptoms are partially explained by chronic stress in everyday life - marital stress and social stress are significantly (positively) associated with loneliness, other stressors and life events are not  • The associations between high-school diploma, group membership and loneliness are explained by social network size (significantly negatively related to loneliness itself), while frequency of contact with social network does not impact loneliness • Relationship quality partially explains the effect of marital and social stress and education on loneliness • Greater network satisfaction and having a spouse as a confidant are negatively linked to loneliness, while not having a spouse as a confidant (but still being married) is not linked to loneliness |
| Hawkley & Kocherginsky (2018) | • US • 2005-2011 • National Social Life, Health and Aging Project • Longitudinal (2 waves) • N=2,261 (aged 57-85, 52.2% female, residing in community) | Risk factors:  • Demographic (age, sex, ethnicity) • Socio-economic (education, household assets, income to needs ratio, working status) • Social (living arrangements, marital status, social network size, proportion of kin, number of close relatives and friends, frequency of socialising with friends and family, attendance at group meetings and in church, family and friend support and strain, spousal support and strain for the married, relationship closeness, spousal closeness for the married) • Health (self-reported, functional limitations)  Loneliness measure: UCLA 3-item scale --> binary (based on mean score) | • Multivariate linear regressions • Logistic regressions | • In cross-sectional analysis, negative associations are found between loneliness and age, being married, higher number of friends, socialising more frequently, self-rated health and family support, and positive associations between loneliness and living alone and family strain • Loneliness is predicted by its lagged values, and by lagged functional limitations, little support from the family and more strain in friendships (but these 3 effects are small), no other variables have a significant effect • Predictors of becoming lonely between the two waves are only functional limitations and less family support • Predictors of recovering from loneliness are only socialising more frequently, having a better self-reported health and less family strain |
| Hawkley et al. (2020) | • US • 2004-2016 • Health and Retirement Study • Longitudinal (7 waves) • N=6,532 (aged 50+, 60.3% female) | Risk factors:  • Negative wealth shock (loss of 75+ % of the net household wealth) • Negative income shock (loss of 75+ % of household income) • Socio-economic (labour force status, net household wealth, household income) • Social (marital status, household size, religious service attendance, spousal and family strain) • Health (insurance, chronic conditions, difficulties with activities of daily life and instrumental activities of daily life - ADLs and IADLs) • Recession  Loneliness measure: UCLA 3-item scale --> 0-6 score | Ordinal logistic fixed-effects models | • Negative wealth shock does not have an impact on loneliness • Negative income shock increases loneliness and the effect is independent of baseline income levels and of life events potentially linked to income loss such as marital dissolution • Role of negative income shock on loneliness is not mediated through health status, religious attendance or relationship quality, but the effect is mitigated if the shock happened during the Great Recession • Having larger household income is linked to less loneliness (but the effect is small), so is having a partner, while living in a household with 2+ members is associated with more loneliness compared to just 2 members • Recession itself, household wealth, labour status, health insurance and health status do not have any effect on loneliness |
| Heshmati et al. (2021) | • US • 2005-2008 • Study of Early Childcare and Youth Development • Cross-section • N=512 (child aged 15+, 50% female child) | Risk factors:  • Child sex • Socio-economic (parental education, family income) • Social (relationship quality between the child and each parent, interparental relationship quality) • Parental mental health (depression)  Loneliness measure: Loneliness and Social Dissatisfaction Questionnaire --> overall score | • Analysis of triadic structures • One-way ANOVA • Hierarchical multiple regressions | • Relationship configurations with one low quality tie between the child and a parent (regardless of the quality of interparental tie) are linked to higher loneliness of the child compared to situation with high quality ties only • When controlling for other covariates, having all high quality ties is linked to lower loneliness compared to when there is at least one low quality tie • Father's education and both parents' depression are linked to higher loneliness of the child • There is no effect of child's sex on their loneliness, and no effect of family income or mother's education either |
| Hoffart et al. (2020) | • Norway • 2020 • Norwegian COVID-19 Mental Health and Adherence project • Cross-section • N=10,061 (aged 18-86, 78% female) | Risk factors:  • Demographic (age, sex and identification with biological sex) • Socio-economic (education, refugee status, employment status) • Social (civil status, number of children) • Presence of psychiatric diagnosis • COVID-19-related (suspicion about being infected, time staying at home and reasons, health anxiety, worry about job and economy, general worry and rumination, coping strategies - time being engaged in activities one would normally not have time to do and time spent experiencing nature)  Loneliness measure: UCLA loneliness scale-8 --> 8-32 score | Hierarchical regression analyses | • Age, male sex, being married, being employed and both coping strategies (doing new things and experiencing nature) are significantly negatively associated with loneliness • Having a psychiatric diagnosis, worrying about job and economy, having health anxiety, and worrying and ruminating in general are all significantly positively associated with loneliness • Being transgender, having higher education, having children and being a refugee are not linked to loneliness when other factors are controlled for • Effect of rumination and general worry reaches a medium size, while effects of being single, having a diagnosis, worrying about job and economy, and doing new things have a small size, and all other effects are negligible |
| Hoffart et al. (2022) | • Norway • 2020 • Norwegian COVID-19 Mental Health and Adherence project • Longitudinal (2 waves) • N=4,936 (aged 18+, 79% female) | Risk factors:  • Demographic (age, sex and identification with biological sex) • Socio-economic (education, refugee status, employment status) • Social (partnership status, living status, having children, emotional support, frequency of face-to-face and virtual social contact, coping behaviours) • Mental health (coping strategies, health anxiety, metacognitive beliefs) • COVID-19-related (home confinement, COVID-19 related changes in employment status, fear of being infected by and dying from COVID-19, worry about job and economy)    Loneliness measure: UCLA loneliness scale-8 --> 8-32 score | Mixed models | • Younger age associated with higher initial levels of loneliness, but also a larger reduction in loneliness over time  • Other demographic and social variables not linked to feelings of loneliness • Changes in health anxiety, worry about job and economy, and negative metacognitive beliefs linked to less reduction in loneliness, while changes in positive metacognitive beliefs without impact on reduction of loneliness • Unhelpful coping strategies linked to more reduction in loneliness over time, but their change linked to less reduction in loneliness over time • Emotional support linked to lower initial levels of loneliness, but also less reduction in it over time, while increases in emotional support over time linked to more reduction in loneliness  • Coping behaviours (and their changes) not associated with loneliness or its changes  • Increase in physical (but not virtual) contact with friends and relatives linked to more reduction in loneliness over time |
| Hu & Gutman (2021) | • UK • 2020 • Understanding Society COVID-19 Study (UK Household Longitudinal Study) • Longitudinal (4 waves) • N=419 (aged 18-25, 70.6% female) | Risk factors:  • Demographic (sex, ethnicity) • Socio-economic (household income, employment status just before the pandemic, education status - being in school just before the pandemic) • Social (emotional support change, living with a partner) • Baseline physical or mental health condition  Loneliness measure: Single item: "How often do you feel lonely?" (hardly ever or never/some of the time/often) --> change in mean score | Growth curve modelling | • Being employed, being in education or having a higher income are associated with lower levels of loneliness • Having a pre-existing mental or physical health condition are positively associated with loneliness • Sex, ethnicity and living with a partner do not impact loneliness • Emotional support protects from loneliness, but only among men |
| Hutten et al. (2022) | • Netherlands (Limburg region) • 2016 • Community Health Services of North and South Limburg survey • Cross-section • N=52,341 (aged 17-101, 53% female) | Risk factors:  • Demographic (age, sex, migration background)  • Socio-economic (education, adequacy of financial resources, employment status)  • Social (living alone, informal caregiving, volunteering, frequency of social contact, social network type)  • Health (physical disabilities, mental health)    Loneliness measure: 11-item de Jong Gierveld scale --> 0-11 score | Linear regression models | • Loneliness increases linearly with age  • Male gender, lower education levels, inadequacy of financial resources, mental health, and limited social contact (especially with friends) are positively associated with loneliness across age groups, with mental health being the strongest correlate of loneliness  • Non-burdensome informal care is linked to lower levels of loneliness across age groups, while informal caregiving experienced as burdensome is linked to higher levels of loneliness  • Having a migration background (especially non-Western) is associated with higher loneliness in all except older adults, living alone in all except young adults, and having physical disabilities in all except middle-aged adults  • Having a paid job is linked to less loneliness only in middle-aged adults, at least when other variables are considered  • Volunteering is not associated with loneliness when other variables are controlled for |
| Hysing et al. (2020) | • Norway • 2014-2018 • Students’ Health and Wellbeing Study • Repeated cross-section (2) • N=13,525 in 2014 (aged <35, full-time students) • N=50,054 in 2018 (aged <35, 69.1% female, full-time students) | Risk factors:  • Demographic (age, sex, migrant status) • Social (relationship status, accommodation status) • Living/studying abroad  Loneliness measure:  • Single item: "In the past two weeks, including today, how much have you been bothered by feeling lonely?" (not at all/a little/quite a bit/extremely) --> binary (top 2 categories collapsed) • In 2018, UCLA 3-item scale --> categories or total score | • Chi-square tests • Logistic regression • ANOVA | • Age displays a curvilinear (U-shaped) relationship with loneliness - the youngest and oldest students have the highest levels of loneliness • Age interacts with sex - female students are lonelier than male students, but the sex difference is more pronounced for younger students • Higher likelihood of being lonely is found in single students, especially men, those living alone or with parents, especially men, among women studying abroad, and among students who are immigrants to Norway |
| Ikizer et al. (2022) | • 41 countries • March-May 2020 • COVIDi-STRESS  Global Survey • Cross-section • N=99,217 (aged 18-110, 72.6% women) | Risk factors:  • Demographic (age, sex, country of residence)  • Socio-economic (education)  • Marital status  • Big Five personality traits    Loneliness measure: 3-item UCLA scale --> score | • Multi-group confirmatory factor analysis  • Multilevel regression  models | • Male gender and higher age associated with lower levels of loneliness  • All personality traits except openness (not significant) and conscientiousness (associated with less loneliness) associated with higher loneliness, especially neuroticism |
| Koelet & de Valk (2016) | • Belgium • 2012-2013 • EUMARR survey • Cross-section • N=338 (aged 30-45, 58% women, married or cohabiting with a Belgian partner) | Risk factors:  • Sex • Social (number of own family and "in-laws" living in Belgium, number of close friends in Belgium and % of these that are "own" friends and that are native Belgians, frequency of contact with local and transnational network of friends and family) • Duration of residence  Loneliness measure: Social loneliness subscale from 11-item de Jong Gierveld scale --> factor score scale | Structural equation modelling | • Migrants with a native partner have higher levels of social loneliness compared to natives with a native partner • Transnational contact with friends and family and share of native friends have no effect on loneliness, but number of local friends and frequency of contact with these impact social loneliness negatively • Having more own family in Belgium is associated with lower loneliness, but not the frequency of contact with them or having more in-laws in Belgium • Social loneliness is lower for those who are in the country for longer (but it is an indirect effect that goes through having more own family in Belgium and having more own local friends) • There is no effect of sex on loneliness |
| Krause (2016) | • US • 2014 • Landmark Spirituality and Health Survey • Cross-section • N=1,774 (aged 18+, 62% women) | Risk factors:  • Demographic (age, sex) • Education • Social (marital status, frequency of attending church, spiritual and emotional support) • Health (self-rated, physical illness) • Humility  Loneliness measure: UCLA 3-item scale --> 3-9 score | Regression analyses | • Not being married is strongly positively linked to loneliness, female sex as well  • Education and age are not linked to loneliness • Church attendance, spiritual support and humility per se do not impact loneliness • Emotional support received does have a strong negative effect on loneliness (and humility is strongly linked to emotional support, so there is an indirect significant effect of humility on loneliness) • Loneliness is linked to reporting more physical health symptoms and worse self-perceived health |
| Kristensen et al. (2021) | • Germany • 2008-2017 • German Ageing Survey • Longitudinal (4 waves) • N=19,187 observations (10,158 individuals) (aged 40+, 50% female, with children) | Risk factors:  • Age • Income • Marital status • Presence of a child in the household • Functional limitations • Perceived housing situation  Loneliness measure: 6-item de Jong Gierveld scale --> 1-4 mean score | Fixed effects linear regression | • Transition to an empty nest does not have an impact on women's loneliness and it decreases loneliness in men, but the effect vanishes in multivariate analyses (the interaction between sex and empty nest is significant only in bivariate analyses) • Being widowed (but not being divorced or living separately) increases loneliness compared to being married among both sexes, but more strongly for men and being single decreases loneliness among women • Being retired decreases loneliness slightly for both sexes compared to being employed • Functional limitations increase loneliness, but the effect is basically null • Age, income and self-rated living situation do not impact loneliness |
| Kung et al. (2022) | • UK • 2006-2020 • UK Biobank study • Cross-section and longitudinal (2 waves)  • N=380,505 (aged 40-70, 52.9% female) for cross-sectional analysis  • N=36,153 (aged 40-70, 49.9% female) for longitudinal analysis | Risk factors:  • Demographic (age, ethnic background)  • Socio-economic (education, household income, financial difficulties, employment status)  • Social (marital status, household composition, family composition)  • Health  • Neighbourhood socio-economic environment  • Recent adverse life events  Loneliness measure: Single item: “Do you often feel lonely?” (yes/no) --> binary | • Linear probability regression model  • Non-linear transitional panel analysis | • Low education levels, low household income, financial difficulties, being unemployed or unable to work due to disability and residing in deprived areas are all associated with higher loneliness  • Being in retirement is associated with lower levels of loneliness, as well as being older, of male gender, being married or cohabiting (especially for men)  • Having children is linked to higher levels of loneliness, while having surviving parents is linked to lower levels of loneliness (especially for women) |
| Lai et al. (2021) | • UK • 2006-2010 • UK Biobank study • Cross-section • N=390,169 (aged 37-73, 52.6% female) | Risk factors:  • Demographic (age, sex) • Socio-economic (education, employment, income) • Social (number of children, phone usage) • Health (smoking, MET minutes of walking per week, BMI, cardio-metabolic risks) • Anxiety/depression • Environmental (residential unit density - 1km and 2km, public transport density, street distance to destinations, street-level movement density, traffic intensity in the nearest road, terrain variability - 500m, residential greenness - 500m)  Loneliness measure: Two single items: "Do you often feel lonely?" (yes/no) and "How often are you able to confide in someone close to you?" (almost daily/2-4 times a week/about once a week/about once a month/once every few months/never or almost never) --> binary (based on both questions combined) | Logistic regression models | • Higher residential density is linked with a higher likelihood of being lonely when controlling for all other covariates (but the effect is very small) • This effect on loneliness is stronger for men than women and for those retired or in employment • Higher likelihood of loneliness is linked to higher density of self-contained flats, while higher density of terraced housing is not linked to likelihood of loneliness, and higher density of detached housing is linked to a lower likelihood of loneliness  • More MET minutes of walking are associated with a lower likelihood of loneliness |
| Lampraki et al. (2022) | • Switzerland • 2020 • NCCR LIVES COVID-19 study • Longitudinal (3 waves) • N=737 (aged 18-81, 66.3% female) | Risk factors:  • Demographic (age, sex) • Socio-economic (education, income adequacy, student status, employment status) • Social (marital status, living alone, frequency of using different communication tools, social contacts and their frequency) • Subjective health  • Pandemic-related (sanitary measures, social distancing measures)  Loneliness measure: 6-item de Jong Gierveld scale --> mean social and emotional loneliness scores | • Correlations • Repeated-measures ANOVA  • Bivariate latent growth-curve model (LGM)  • Multilevel linear models | • Better subjective health and its improvements are linked to lower levels of social and emotional loneliness, and are the strongest predictors  • Being younger and living alone are associated with more emotional, but not social loneliness, while higher education is only linked to higher social loneliness • Social distancing and sanitary measures, and number of friends or relatives contacted are not associated with feelings of loneliness • Traditional ways of communication or communication through video are unrelated to both types of loneliness, while communication through social media is associated with higher social and especially emotional loneliness • Higher number of relatives and especially friends as SOS contacts is linked to lower levels of social and emotional loneliness, while in terms of changes over time the decrease in number of friends as SOS contacts is linked to higher social loneliness and the decrease in number of friends considered confidants is linked to higher emotional loneliness  • Higher frequency of social interactions and its increase over time is linked to lower social, but not emotional loneliness |
| Lampraki et al. (2019) | • Switzerland • Year not reported • LIVES Intimate Partner Loss study • Cross-section • N=1,719 (aged 40-92, 59.9% female, married and never divorced or divorced/separated in the past 5 years) | Risk factors:  • Demographic (age, sex) • Income adequacy • Social (availability of social resources - children, new partner (only for the divorced), someone to count on, multiple important group memberships (MIGM) score) • Subjective health • Psychological (personality traits, self-continuity)  Loneliness measure: Social loneliness subscale of 11-item de Jong Gierveld scale --> mean score | • ANOVA • Regression analyses | • Divorced people are lonelier than the married ones in general, but there is no difference in loneliness between those divorced for a short or a long time • Having children and openness do not impact loneliness • Better subjective health, extroversion and higher income are linked to lower loneliness (income less so for shortly divorced), having someone to count on as well (but it is not asked to the married) • Being a women is linked to lower loneliness in long-term divorced and married, and so is MIGM and self-continuity, while age is linked to lower loneliness only in the married group, having a new partner only among the long-term divorced (not for short-term divorced, not asked to the married), conscientiousness only for the married, agreeableness for the married and short-term divorced • Neuroticism is linked to higher loneliness only for the short-term divorced |
| Lasgaard et al. (2016) | • Denmark • 2013 • Danish National Health Survey • Cross-section • N=33,285 (aged 16-102, 50.4% women) | Risk factors:  • Demographic (age, sex, ethnicity) • Socio-economic (educational level, employment status) • Social (cohabitation and partner status, parental status) • Health (contacts with GP, long-term health conditions, hospital admissions, mental health disorders, psychiatric treatment) • Housing status (owner/tenant) • Area (urbanisation, residential area - deprived or not)  Loneliness measure: 3-item Loneliness Scale --> binary moderate (score 5/7 or higher) and binary severe (score 7/7) | Binary and multinomial logistic regressions | • There is a U-shaped relationship between loneliness and age, more pronounced for moderate loneliness than for severe loneliness • Being a woman, of a non-Danish ethnicity, being unemployed or receiving disability pension, living alone (as divorced/widowed/never married), being a tenant, living in a rural or a deprived area, having chronic conditions, psychiatric treatment or mental health disorders are all positively linked to the likelihood of reporting both moderate and severe loneliness • Lower education and being a student are only linked to the likelihood of severe loneliness, while number of GP visits only to the likelihood of moderate loneliness • Being a parent, being in retirement and number of hospital admissions are not linked to the likelihood of feeling lonely when other factors controlled for (but there is a link in bivariate analysis) • Ethnicity, unemployment, disability pension and mental health conditions are universal predictors for all ages • Sex, education and area deprivation impact the likelihood of being lonely only among young adults, living alone in all age groups except old adults, and being a tenant during early to middle adulthood |
| Lepinteur et al. (2022) | • Germany • 2017-2020 • German Socio-Economic Panel Study (SOEP)  • SOEP COVID-19 study • Longitudinal (2 waves) • N=5,446 (aged 18+, 52% female) | Risk factors:  • Demographic (age, sex) • Socio-economic (income, education, work status) • Social (partner status, family size, presence of a child in the household, household size in m^2^)  • Macro-region in Germany (East/West) • Number of health conditions before COVID-19 pandemic  Loneliness measure: UCLA 3-item scale --> 3-15 score | Difference-in-differences analysis | • Women found to be lonelier than men before and during the COVID-19 pandemic  • Gender gap in loneliness increased significantly during the COVID-19 pandemic compared to before |
| Li & Wang (2020) | • UK • 2020 • Understanding Society COVID-19 Study (UK Household Longitudinal Study) • Cross-section • N=15,530 (aged 18+) | Risk factors:  • Demographic (age, sex) • Employment status • Living alone • Country of residence (within UK) • Presence of current and past COVID-19 symptoms  Loneliness measure: Single item: "In the last 4 weeks, how often did you feel lonely?" (hardly ever or never/some of the time/often) --> categories | • Univariate analysis • Ordered logistic regression models | • In bivariate analysis, often feeling lonely is more frequent among those who currently have COVID-19 symptoms and a bit more among those who ever had symptoms, more frequent among females, young people and those living without a partner, and slightly more among those unemployed, while region of the UK doesn't have any impact on loneliness • In multivariate analysis, these effects are confirmed, with the effect of living without a partner being the strongest, followed by age and sex |
| Lim et al. (2022) | • Australia, UK, US • 2020 • Longitudinal • N=1,562 (N=701 for Australia, N=483 for UK, N=378 for US, aged 18-91, 84.2% female) | Risk factors:  • Demographic (age, sex) • Socio-economic (employment status, financial status) • Social (living alone, carer, have children under 16 years) • Pandemic-related (severity of social restrictions, personal and overall exposure to COVID-19)    Loneliness measure: UCLA 20-item scale --> loneliness score | Multivariate latent growth curve model (MLGC) | • Being young, unemployed, with lower income, having a child under 16 years, being a carer and living alone are all associated with higher initial levels of loneliness, but none of them predicts changes in loneliness over time • Sex and pandemic-related variables do not have a statistically significant effect on loneliness |
| Lin (2023) | • Canada • 2021 • Canadian Perspective Survey Series – Substance Use and Stigma during the Pandemic (CPSS6-COVID) • Cross-section • N=3,722 (aged 15+, 53.8% female) | Risk factors:  • Demographic (age, sex, migration status) • Socio-economic (education, precarious employment due to COVID-19)  • Social (marital status, household size, social network size, social participation) • Environmental (urban or rural area, housing type) • Health behaviours (binge drinking, opioid use, cannabis use) • Mental health (self-rated, perceived life stress, change compared to before COVID-19, mental health help seeking)    Loneliness measure: UCLA 3-item scale (adapted) --> binary (score 7 or more) | • Chi-square tests • Binary logistic regressions  • Classification and regression tree (CART) | • Being young, female, being unemployed due to COVID-19, living alone, having a smaller social network, and engaging in binge drinking and cannabis use are associated with higher levels of loneliness • Lower education is linked to higher loneliness only for women, and the effect of COVID-19-related unemployment and cannabis use are driven by the female sample as well  • Being a migrant and using opioids is associated with higher loneliness only in the male sample, which also drives the effect of living alone  • Urban vs rural area, housing type, social participation and marital status are unrelated to feelings of loneliness  • According to CART, job precarity due to COVID-19 is the strongest predictor of loneliness |
| Lodder et al. (2015) | • Netherlands • 2012 • Cross-section • N=1,172 (Mage = 12.8, 50.9% female, in the first grade of Dutch secondary school) | Risk factors:  • Sex • Social (number of friends in the class - unilateral-given, unilateral-received, reciprocal, name of best friend in the class and quality of the relationship, quality of friendships in the network) • Psychological (depressive symptoms, social anxiety)  Loneliness measure: Loneliness in Peer Relations subscale of the Louvain Loneliness and Aloneness Scale for Children and Adolescents --> overall score | • Hierarchical multiple regression analysis • Logistic regressions • Actor-Partner Interdependence • Model for Indistinguishable Dyads | • Girls are lonelier than boys • Network friendship quality is negatively linked to loneliness and also friendship quantity (number of reciprocal and unilateral-received friendships, but not unilateral-given friendships) • Loneliness is related to a lower likelihood of having a best friend |
| Luhmann & Hawkley (2016) | • Germany • 2013 • German Socio-Economic Panel Study • Cross-section • N=16,132 (aged 18-103, 53% female) | Risk factors:  • Demographic (age, sex)  • Socio-economic (income, education, work status) • Social (living arrangements - household size, having children in the household, relationship status, social engagement - political, religious, volunteering, number of friends, contact frequency - face-to-face contact with friends, relatives, contact with people abroad, contact through social networks) • Functional limitations  Loneliness measure: UCLA 3-item scale --> standardised score | • Locally weighted scatterplot smoothing • Regression analysis | • Age distribution of loneliness has two peaks (at around ages 30 and 60) and two dips (around ages 40 and 75) and a steady increase after age 75  • Female sex, lower income, bigger household size, working full time, having accomplished more years of education, being single, having functional limitations, lower number of friends, less face-to-face contact and more frequent use of social networks predict loneliness  • More education, being in full-time employment and living with others are protective factors of loneliness in bivariate analysis, but they are confounded with income and their effect on loneliness flips sign if income and other factors are controlled for • The effects of income, work status, relationship status and household size on loneliness interact with age • Living with children, engaging in political, religious or volunteer activities and contact with relatives abroad do not impact loneliness |
| Lykes & Kemmelmeier (2013) | • 12/22 European countries (study 1/study 2) • 1992/2006 (study 1/study 2) • Eurobarometer 37.2 (study 1) • European Social Survey (study 2) • Multiple cross-sections (2) • N=3,902 (aged 60-85, 57% female) - study 1 • N=38,867 (aged 14-101, 53% female) - study 2 | Risk factors:  • Demographic (age, sex) • Having a paid job (study 2) • Social (marital status, household size, frequency of contact with family and friends (study 1), frequency of meeting socially with friends, relatives and colleagues (study 2), social activity relative to others of the same age (study 2), having someone to discuss personal matters with (study 2), perceiving being cared for by some other people (study 2)) • Health (long-term illness (study 1), receiving assistance because of limitations in daily activities (study 1), subjective (study 2)) • Community size • Individualism of the country  Loneliness measure:  • Single item (study 1): "Do you feel lonely often, occasionally, or never?" --> categories • Single item (study 2): "How much of the time during the past week you felt lonely?" (none or almost none of the time/some of the time/most of the time/all or almost all of the time) --> categories | Generalised linear mixed models | • In both studies, individualistic countries have lower levels of loneliness • In study 1, age, sex and community size do not have an impact on loneliness • In study 1, being single/divorced/widowed is linked to increased loneliness (widowhood the most strongly), living alone, having long-term illness and receiving assistance as well (this last one even more so in individualistic countries) • In study 1, more frequent contact with family and friends is associated with lower loneliness, but the effect is moderated by individualism - contact with family has a stronger impact in collectivist countries, while contact with friends is more important in individualistic countries • In study 2, age, better subjective health, having a paid job, meeting more socially (also relative to others the same age), having a confidant and people who care are all negatively linked to loneliness, with having a confidant being more important for preventing loneliness in individualistic societies • In study 2, being single/divorced/widowed, being a female, living alone and living in bigger communities are positively linked to loneliness |
| MacDonald et al. (2020) | • Netherlands • 2004 • Netherlands Twin Register • Cross-section • N=8,356 (aged 17-97, 54.5% women) | Risk factors:  • Demographic (age, sex) • Frequency of leisure activities (4 general, 5 specific) • Degree of urbanisation  Loneliness measure: 3-item R-UCLA scale --> 3-9 score | • One-way ANOVA • Regression analysis | • There is significantly higher loneliness among those who live in heavily urbanised areas compared to non-urban areas, but the effect is small • Relationship between age and loneliness is U-shaped • Sex has no impact on loneliness • All social activities lead to lower loneliness, except for listening to music and computer activities (that are linked to higher loneliness), though all positive and negative relationships are small and they are significant only for going to nature/sightseeing/zoo/amusement park, participating in neighbourhood programs/hobby or social clubs/professional organisations and computer activities, not for others (going to movies/theatre/concerts/museums, going to cafes/restaurants/going dancing, physical or mind sports, reading, listening to music and watching TV) |
| Madsen et al. (2016) | • Denmark • 2014 • Health Behaviour in School-aged Children Study • Cross-section • N=4,383 (aged 11-15, 51.2% female) | Risk factors:  • Demographic (age, sex, migrant background) • Family occupational class • School context (member of ethnic majority in class, size of own ethnic group in class - absolute and relative, Simpson's Index of Diversity - measure of ethnic diversity in a class)  Loneliness measure: Single item: "Do you feel lonely?" (yes, very often/yes, often/yes, sometimes/no) --> binary (top 2 categories collapsed) | Multilevel logistic regression analyses | • Loneliness is higher among girls compared to boys and increases with age • Membership in an ethnic minority group in class is linked to a higher likelihood of feeling lonely • Both absolute and relative size of own ethnic group in class impact the likelihood of being lonely - it is higher the lower the size • There is no relationship between the likelihood of being lonely and ethnic diversity in class |
| Marquez et al. (2022) | • UK • 2017-2019 • Understanding Society • Cross-section • N=6,503 (aged 16-24, 55.5% female) | Risk factors:  • Demographic (age, sex, sexual orientation, ethnic group, country of residence)  • Subjective financial situation  • Social (religion, close friends – also of the same age, race and geographical area, going out with friends, time spent with friends on social media)  • Health (self-reported, mental health, long-standing illness)  • Life satisfaction  • Environment (geographic region, urban or rural area, perceived neighbourhood quality, community type)    Loneliness measure: UCLA 3-item scale --> 0-6 score | • Bivariate analyses  • Nested multilevel models | • Not being heterosexual and worse mental well-being are associated with higher loneliness  • Being of minority ethnic group, better self-reported health and life satisfaction, number of friends and especially going out with them when one feels like it, higher perceived neighbourhood quality and a greater sense of belonging to their communities are associated with less loneliness  • Gender, urban or rural area, subjective financial situation, belonging to a religion, long-standing illness or disability, and time spent on social media are not significantly associated with loneliness after controlling for other variables |
| Milicev et al. (2022) | • UK • 2017-2020 • Understanding Society Survey • Longitudinal (7 waves) • N=3,475 (aged 16-94, 58.2% female) | Risk factors:  • Demographic (age, sex) • Social (relationship status, friends of similar age and ethnicity, number of close friends, amount of time on social media with friends) • Health (medical condition requiring shielding during COVID-19)  • Environmental (neighbourhood quality, geographic region within UK, urban or rural area)    Loneliness measure: Single item: "How often do you feel lonely?" (hardly ever or never/some of the time/often) --> score | Multivariate latent growth curve model (MLGC) | • Before the pandemic, age, being female, having bad health, few friends of the same age or ethnicity, or few close friends, being single and living in a bad quality neighbourhood were associated with higher initial levels of loneliness • Before the pandemic, the amount of time spent with friends on social media, living in an urban context and the geographical region were unrelated to loneliness levels • Change in loneliness during the pandemic (compared to before) was predicted by having a medical condition requiring shielding and the number of close friends • No other variables were linked to changes in loneliness during COVID-19 |
| Moens et al. (2021) | • Belgium • 2019 • Cross-section • N=1,358 (employees in private sector) | Risk factors:  • Demographic (age, sex) • Education level • Job-related characteristics (type of contract - permanent/temporary, full/part-time, job satisfaction, supervisory position, interaction outside organisation, job complexity, specialised knowledge requirements, job tenure, work from home days) • Social (religion, relationship status, having children, living alone) • Psychological (HEXACO personality traits, satisfaction with health/social/family life) • Place of residence (rural/urban)  Loneliness measure: UCLA 4-item scale (adapted to a work setting) --> standardised score | Mediation analysis | • Loneliness is higher among temporary workers compared to permanent workers • Loneliness partially mediates the negative relationship between temporary work and job satisfaction |
| Nicolaisen & Thorsen (2014) | • Norway • 2007-2008 • Life Course, Generations and Gender survey • Cross-section • N=14,743 (aged 18-81, 50.7% female) | Risk factors:  • Demographic (age, sex)  • Partner and living status  • Subjective health  Loneliness measure:  • Single item: "Do you feel lonely?" (often/sometimes/seldom/never) --> binary (top 2 categories collapsed) • 6-item de Jong Gierveld scale --> binary (score 2 or more) | Blockwise multiple regressions | • If indirect measure is used, there is a positive linear trend between age and loneliness, while a U-shaped pattern is found with the direct measure (the oldest and the youngest age group are the most lonely) • Women are found to be lonelier if the direct measure is used, while men are lonelier in the two youngest groups if indirect measure is used (men are more socially lonely in all groups and women are more emotionally lonely in the two oldest groups) • With the indirect measure, worse health is the most important predictor of loneliness, followed by not having a partner, male sex and increasing age • With the direct measure, not having a partner is the strongest predictor of loneliness, then bad health, female sex and old age |
| Nicolaisen & Thorsen (2014b) | • Norway • 2002-2008 • Norwegian study of life course, ageing and generations • Longitudinal (2 waves) • N=3,750 (aged 40-80, 51% female) | Risk factors:  • Age • Education • Partner status (level and change) • Subjective health (level and change) • Adverse life events during childhood (conflictual relationship between parents, being bullied for a long period of time, economic problems at home)  Loneliness measure: Single item: "Do you feel lonely?" (often/sometimes/seldom/never) --> binary (top 2 categories collapsed) and binary change | • Chi-square tests • Cross-sectional multivariate analysis • Multivariate logistic regression | • Age, conflicts between parents and education are not related to the likelihood of loneliness cross-sectionally in general, with the exception of conflicts between parents being important for the loneliness of men in older age • Female sex is related to a higher likelihood of feeling lonely in the older but not the middle-aged adults • Not being married or being divorced or widowed, and being in poor health are strongly linked to a higher likelihood of being lonely (partner status in general has the strongest effects), so are economic problems in household in childhood (except for older men) and having been bullied only for men • Death of the partner is the most important predictor of the onset of loneliness, while breakup has an effect only for women • Age, sex, education and changes in health status do not predict the onset of loneliness |
| Nicolaisen & Thorsen (2017) | • Norway • 2007-2008 • Norwegian Life Course, Gender and Generations survey • Cross-section • N=14,725 (aged 18-79, 50.6% women) | Risk factors:  • Demographic (age, sex) • Socio-economic (education, employment) • Social (partner status, frequency of contact with friends, having confidant friends, aspirations for contact with friends - satisfaction with the level of contact and wanting more contact) • Subjective health status  Loneliness measure: Single item: "Do you feel lonely?" (often/sometimes/seldom/never) --> binary (top 2 categories collapsed) | • Chi-square tests • Bivariate logistic regressions • Hierarchical multivariate logistic regressions | • The relationship between age and loneliness displays a slight U-shape • Being a female, not having a partner and being in poor health are linked to a higher likelihood of being lonely in all age groups - marital status is the most important predictor • Lower education is linked to a higher likelihood of loneliness only in the youngest age group • Not being employed and not having confidants are associated with the likelihood of being lonely in the two middle-aged groups • Low contact with friends is linked to a higher likelihood of being lonely in all age groups except the oldest one, but when contact aspirations are controlled for the effect disappears • Aspirations for (satisfaction with) contact are important for the likelihood of being lonely in all age groups - dissatisfaction with the level of contact with friends is one of the strongest predictors of loneliness |
| Niedzwiedz et al. (2016) | • 14 European countries • 2013 • Survey of Health, Ageing and Retirement in Europe • Cross-section • N=29,795 (aged 65+, 55.2% female, not in the paid labour force) | Risk factors:  • Demographic (age, sex, migrant status) • Socio-economic (self-reported household wealth, education) • Social (marital status, household size, frequency of contact with children, frequency of social participation in voluntary/charity work, education/training course, sport/social/other club and political/community organisations) • Functional limitations  Loneliness measure: 3-item Revised UCLA scale --> binary (based on country-specific quartiles) | Multilevel logistic regression models | • Loneliness is more likely among those with lower wealth • Social participation is linked to a lower likelihood of loneliness - it doesn’t mediate the relationship between wealth and likelihood of loneliness, but it moderates it (the link between wealth and likelihood of loneliness is stronger for those who participate socially less, especially among men) • Women are lonelier than men, the more so the less they participate in social activities • Participating in education/training has no effect on the likelihood of loneliness, in political/community organisations has a weak effect, while the effect is stronger for sports and other clubs and voluntary work • Higher education is linked to a lower likelihood of feeling lonely only among women, household size and frequency of seeing children are linked to a lower likelihood of feeling lonely for both sexes, while higher age, being an immigrant, having functional limitations and being divorced, widowed or never married are associated with a higher likelihood of being lonely for both sexes |
| Nyqvist et al. (2021) | • Sweden and Finland  • 2010-2016  • Gerontological Regional Database study  • Longitudinal (2 waves)  • N=4,269 (aged 65+, 53.6% female) | Risk factors:  • Demographic (age, sex)  • Socio-economic (education, making ends meet)  • Social (civil status, frequency of social contract, trust in friends and neighbours, number of confidants, participation in voluntary organisations)  • Health (self-rated health, depression, instrumental and personal activities of daily living)  Loneliness measure: Single item: “Do you suffer from loneliness?” (yes/no) --> binary | • Pearson’s chi-square tests  • Stepwise logistic regression models | • Age, sex, education, financial situation (all at baseline) and any of the social variables (at baseline or their change over time) do not predict loneliness at a later date  • Being widowed or divorced at baseline and becoming widowed are risk factors for loneliness at a later date  • Poor self-rated health at baseline (but not its change) and onset of depression are also risk factors for loneliness |
| Nyqvist et al. (2019) | • 20 European countries • 2014 • European Social Survey  • Cross-section • N=11,389 (aged 60+, 49-59% female depending on welfare regime) | Risk factors:  • Demographic (age, sex) • Education • Level of social integration (marital status, frequency of meeting socially, number of confidants) • Quality of living conditions (self-rated health, perceived safety in the neighbourhood, household size) • Welfare regime (Anglo-Saxon/Continental/Eastern Europe/Nordic/Southern)  Loneliness measure: Single item: “How much of the time during past week did you feel lonely?” (none or almost none of the time/some of the time/most of the time/almost all of the time) --> binary (absence of loneliness against all other categories) | • Chi-square tests • Multivariate logistic regressions | • Absence of loneliness is more common in the Northern, Anglo-Saxon and Continental regimes, and less common in Southern and Eastern European regimes • Welfare regimes improve the model fit, but individual-level features explain most of the variation - the strongest predictors of absence of loneliness are bigger household size and being married • Being in good health is linked to a higher likelihood of absence of loneliness in all regimes except the Southern • Living in a 2-person household is a strong predictor of absence of loneliness across regimes, but in a 3+ person household only in Southern, Eastern European and Continental regimes • Feeling of safety is only relevant for the absence of loneliness in Continental regime • Being married is linked to higher likelihood of absence of loneliness across regimes • Frequent social contacts are only relevant for absence of loneliness in Anglo-Saxon, Southern and Eastern regimes • Larger number of confidants is linked to a higher likelihood of absence of loneliness in all but Northern regime |
| Nyqvist et al. (2016) | • Finland • 2011 • Finnish National Institute for Health and Welfare's survey • Cross-section • N=4,618 (aged 15-80) | Risk factors:  • Demographic (age, sex) • Education • Marital status • Language • Structural social capital (frequency of contact with friends and neighbours, activity in associations) • Cognitive social capital (trust in others, feeling of belonging in neighbourhood)  Loneliness measure: Single item: "Do you feel lonely?" (often/sometimes/seldom/never) --> binary (top 2 categories collapsed) | • Pearson's chi-square tests • Logistic regressions | • Descriptively, loneliness is more common in younger age groups • In multivariate analysis, only mistrust is a universal predictor of loneliness across age groups • Strong feeling of neighbourhood belonging is associated with a lower likelihood of being lonely in all ages except in 30-49 age category, while being active in organisations is associated with a lower likelihood of loneliness in this age group only • Low frequency of contact with friends is linked to a higher likelihood of being lonely in all age groups except the oldest one • Low frequency of contact with neighbours is linked to a higher likelihood of loneliness only for the youngest and the oldest adults |
| Ormstad et al. (2020) | • Norway • 2002-2008 • Norwegian study of life course, ageing and generations • Longitudinal (2 waves) • N=935 (aged 60-79, 44.8% female, not lonely at baseline) | Risk factors:  • Demographic (age, sex) • Living with a partner • Psychological (Big Five personality traits, mental health, depression, anxiety)  Loneliness measure: Single item: "Have you felt lonely during the past week?" (never/seldom/sometimes/often) --> binary (top 2 categories collapsed) | • Chi-square tests • Multivariate logistic regressions | • High agreeableness is linked to a higher likelihood of becoming lonely in women and lower likelihood of becoming lonely in men • High conscientiousness decreases the risk of becoming lonely for men, but there is no effect for women • High neuroticism increases risk of becoming lonely among men, but there is no effect for women • There is no link between openness and extroversion and becoming lonely |
| Pagan (2020) | • Germany • 2013-2017 • German Socio-Economic Panel • Cross-section • N=42,569 (aged 16+, 53.6% female) | Risk factors:  • Demographic (age, sex, nationality) • Socio-economic (education, household income, employment status) • Social (marital status, having children in the household, household size, relational time index - mean of frequency of contact with friends, family, neighbours, social networks use, frequency of participation in volunteering, sports, cultural and religious activities) • Disability status (no/moderate/severe) • Region  Loneliness measure: UCLA 3-item scale --> 0-4 mean score | • Locally weighted scatterplot smoothing • OLS regressions | • Disability is a strong predictor of loneliness • Women are lonelier than men, the more so the more disability increases • For men the strength of the relationship between disability and loneliness decreases with age, the same holds for moderate disability and loneliness for women, while the relationship between severe disability and loneliness strengthens with age for women  • More education and having children in the household have negative, but very small effects on loneliness for both sexes, while being German, having a higher income, being employed and having more frequent social interactions all have negative and bigger effects on loneliness (the last one more for women, higher income more so for the 2 middle-aged categories) • Being single and living without a partner have a positive effect on loneliness, the former especially for men • Household size doesn't impact loneliness |
| Pan et al. (2023) | • UK  • 2018  • BBC Loneliness Experiment  • Cross-section  • N=2,164 (aged 55+, 62-64% female, 50% non-migrants, 11% cultural migrants, 39% similar-culture migrants) | Risk factors:  • Demographic (age, sex  • Socio-economic (education, employment status, income)  • Social (environment – social capital, discrimination and ageism, situation – marital status, having children and relational mobility)  • Self-reported health  • Coping strategies for loneliness  Loneliness measure: UCLA 4-item scale --> score | • One-way ANOVA  • Chi-square tests  • Multiple linear regressions | • Being married or cohabitating and relational mobility are correlated with lower levels of loneliness for non-migrants and similar-culture migrants but not among cultural migrants, while income is a protective factor for loneliness only among the two groups of migrants but not for non-migrants  • High social capital, active coping strategies and better self-reported health are protective factors for loneliness among migrants and non-migrants, while discrimination, ageism and not being aware of coping strategies have a positive association with loneliness for all groups of population  • Age, sex, education, employment status, having children and passive coping strategies are unrelated to loneliness in all groups of population |
| Penning et al. (2022) | • Canada  • 2007-2018  • General Social Surveys  • Cross-section  • N=49,892 (aged 45+, 48.3-52.6% female) | Risk factors:  • Demographic (age, sex, immigrant status)  • Socio-economic (education, employment status, household income)  • Social (childlessness, partner status, religion)  • Health (subjective, health condition)  • Region of residence  Loneliness measure: 6-item de Jong Gierveld loneliness scale --> overall, social and emotional loneliness 1-3 mean score | Linear regressions | • There is a positive association between being childless and overall and social loneliness, but not emotional loneliness, and this link is more pronounced for men, older adults and the widowed  • Women report lower levels of both overall and social loneliness, but do not differ from men in terms of emotional loneliness  • Older age groups and married individuals report lower levels of all types of loneliness, with the only exception being the same levels of social loneliness for the married and the widowed  • Being foreign born, having lower household income and being in worse health are linked to higher levels of all types of loneliness  • Belonging to a religion is associated with higher emotional and lower social loneliness and the opposite is true for having higher education  • Being in retirement (compared to being employed) is linked to lower levels of overall and social loneliness, but not emotional loneliness |
| Rapolienė & Aartsen (2022) | • 28 European countries and Israel  • 2012  • European Social Survey  • Cross-section  • 12,042 (aged 65+) | Risk factors:  • Demographic (age, sex)  • Socio-economic (income adequacy, education)  • Social (social engagement, marital status, living alone)  • Psychological (generalised trust, trust in the system)  • Health limitations  • Country-level (post-totalitarian country indicator)  Loneliness measure: Single item: “How much of the time during the past week…you felt lonely?” (none or almost none of the time/some of the time/most of the time/all or almost all of the time) --> binary (top 2 categories collapsed) | Path model: measurement part (estimation of the latent variables) and structural part (linear and logistic regressions to estimate the direct and indirect effects of risk factors) | • Likelihood of being lonely is higher in post-totalitarian European countries, but the effect vanishes once socio-demographic and other individual characteristics are accounted for  • Trust is not directly related to loneliness, but explains a part of the link between lower social engagement and higher loneliness  • Being widowed or divorced are the strongest predictors of loneliness and also health limitations and income inadequacy are significantly related to loneliness  • Education, gender and age are not associated with loneliness |
| Rote et al. (2013) | • US • 2005-2006 • National Social Life, Health, and Aging Project (NSHAP) • Cross-section • N=2,165 (aged 57-85, 52% female) | Risk factors:  • Demographic (age, sex, race/ethnicity) • Socio-economic (education, employment status, income) • Social (marital status, number of children/grandchildren, religious attendance, social integration - product of social network size and frequency of contact, social support, frequency of secular meetings) • Health (depression, chronic illness, disability, visual or hearing impairment) • Attractiveness  Loneliness measure: R-UCLA 3-item scale and single item about frequency of feeling lonely in the past week (rarely/some of the time/occasionally/most of the time) --> standardised score (both measures merged) | • OLS regressions • Sobel test for mediation | • Religious attendance is directly negatively associated with loneliness, but when controls are added the relationship disappears • Religious attendance has a positive effect on social integration and social support (and these two are also positively associated), social integration has a weak negative relationship with loneliness (and when social support is added to the model, it becomes insignificant) and social support has a strong negative relationship with loneliness --> hence, religious attendance impacts loneliness indirectly through both social support and social integration • Depression has a strong positive relationship with loneliness |
| Ruffolo et al. (2021) | • Norway, US, UK, Australia • 2020 • Survey about COVID-19 • Cross-section • N=3,810 (aged 18+, 80% female) | Risk factors:  • Demographic (age, sex) • Socio-economic (education, employment status, work setting) • Social (cohabitation - with partner/spouse or not, living with children) • Living area  Loneliness measure: 6-item de Jong Gierveld scale --> scores for overall, social and emotional loneliness | • ANOVA • t-tests • Chi-square tests | • Employed people report lower loneliness than unemployed people (for the US this is true only for emotional loneliness) • Among the employed, overall and social loneliness are lower in Norway than in other countries, and emotional loneliness is lower in Norway and Australia compared to the US and the UK (and it is lower in the US than UK)  • Higher educational level among the employed is associated with lower loneliness • Among those employed, those furloughed/laid-off have higher levels of loneliness than those working remotely, or with little change to their work setting |
| Rumas et al. (2021) | • Canada • 2020 • Study about coping during COVID-19 • Longitudinal (2 waves) • N=797 (Mage = 32.2, 54.6% female) - at baseline • N=395 (Mage = 33.7, 55.7% female) - at follow-up | Risk factors:  • Demographic (age, sex, ethnicity) • Socio-economic (income, education) • Social (social network score, social contact, partner status, living alone) • Physical and mental health diagnoses • Quality of life • Beliefs about social distancing  Loneliness measure: UCLA 20-item loneliness scale --> overall score | Multivariate regression analyses | • Age and social network size are negatively associated with loneliness cross-sectionally and longitudinally • More physical and mental health disorders are associated positively with current and future loneliness (mental health is also the only factor that remains significant in longitudinal analysis if baseline loneliness is controlled for) • Frequency of virtual contact at baseline is linked to higher loneliness at follow-up (but not at baseline), while in-person contact or frequency of leaving home have no effect on loneliness cross-sectionally or longitudinally • There is no effect of sex, ethnicity, income, education, partner status or living alone on current or future loneliness • Loneliness is strongly associated with lower quality of life as well (loneliness is used as a predictor for cross-sectional and longitudinal analyses) |
| Shovestul et al. (2020) | • US • 2017-2019 • Survey on TestMyBrain.org • Cross-section • N=4,536 (aged 10-100, 63% female) | Risk factors:  • Person-related (age, sex, race/ethnicity) • Place-related (population and median household income of given census area) • PersonXplace (% of people with the same age/sex/ethnicity/race in given census area)  Loneliness measure: UCLA 3-item scale --> 3-9 score | Hierarchical linear regressions | • Age and loneliness have a non-linear relationship - loneliness increases steeply until the age of ~19 years and decreases slowly afterwards • Median household income in the census area is negatively linked to loneliness  • Sex, race/ethnicity, population density and age/sex/race/ethnic density in the census area do not impact loneliness |
| Smale et al. (2022) | • Canada  • 2019  • Nova Scotia Quality of Life Survey  • Cross-section  • N=12,826 (aged 16+, 58.2% female) | Risk factors:  • Demographic (age, sex)  • Socio-economic (employment status, household income, self-reported financial situation)  • Social (marital status, number of close friends, number of neighbours from whom favours could be requested, leisure participation, use of community recreation and cultural facilities)  • Health (self-reported physical and mental health, chronic illness or disability)  • Environment (geographical location, perceived accessibility to recreation facilities, sense of belonging to the community)  Loneliness measure: UCLA 3-item scale --> mean score | • Independent sample t-tests  • ANOVA  • Pearson correlation coefficients  • Hierarchical regression analysis | • In the overall population, women, younger adults, those in paid employment and with lower income report significantly higher feelings of loneliness, while those who are married report less feelings of loneliness compared to their counterparts, and there is no significant difference in loneliness between those living in urban or rural areas  • Among older adults, health (physical and mental) and smaller social networks are significant risk factors for loneliness, while sense of belonging to the community and being married are protective against loneliness, and age, sex, income and employment are not associated with loneliness when controlling for other factors |
| Stavrova et al. (2022) | • *Study 1*: Netherlands, 2012, Longitudinal Internet Studies for the Social Sciences, Cross-section, N=2,701 (aged 19-90, 47.6% female)  • *Study 2*: Amazon Mechanical Turk (online panel), Longitudinal – 7-day diary study, N=460 (M(age)=36.5, 47.4% female)  • *Study 4*: UK, Prolific  Academic, Longitudinal – 7-day diary study, N varies between 265 (M(age)=34.3, 74%  female) and 200 (M(age)=34, 77% female) | • *Study 1*: Risk factors: demographic (age, sex), socio-economic (education, employment status, household income), social (co-habiting with a partner, number of children), psychological (self-control, Big Five personality traits); Loneliness measure: UCLA 6-item scale --> mean score  • *Study 2*: Risk factors: Psychological (life satisfaction, presence for meaning and search for meaning, self-control, daily self-control failure, daily happiness, daily meaning, daily self-esteem, daily sadness, daily sense of true self); Loneliness measure: single item: extent of feeling lonely in the past 24 hours  • *Study 4*: Risk factors: psychological (trait self-control, trait-perceived ostracism, momentary ostracism experience, momentary self-control failure); Loneliness measure: UCLA 20-item scale --> score; single item: extent of feeling lonely in the past hour | • *Study 1*: Multilevel regression models  • *Study 2*: Correlations, multilevel regressions  • *Study 4*: Correlations, multilevel regressions | • *Study 1*: Lower self-control is associated with higher loneliness, and so is openness and being unemployed, while extroversion, agreeableness, emotional stability and having a partner are associated with less loneliness, and conscientiousness, age, sex, number of children, education, income and being a student or a housekeeper are not related to loneliness  • *Study 2*: Individuals with higher trait self-control tend to experience less loneliness on a day-to-day basis and daily failures at resisting temptations are associated with more daily loneliness (the latter finding is confirmed by longitudinal data, while the opposite relationship is not found); life satisfaction, daily true self (also longitudinally), daily happiness and daily self-esteem are associated with less daily loneliness, while search for meaning and daily sadness (also longitudinally) with more daily loneliness  • *Study 4*: Low trait self-control and high trait-perceived ostracism are associated with more loneliness, with the latter mediating the effect of the former on loneliness; momentary self-control failures are associated with more momentary loneliness only if they bring about negative consequences for others, with the effect being confirmed by longitudinal analyses and mediated by perceived ostracism |
| Sundström et al. (2009) | • 11 European countries (+Israel) • 2004-2006 • Survey of Health, Ageing and Retirement in Europe • Cross-section • N=8,787 (aged 65-104, 54% female, non-institutionalised) | Risk factors:  • Demographic (age, sex) • Education • Living arrangements • Subjective health  Loneliness measure: Single item: "How often have you experienced the feeling of loneliness over the last week?" (almost all of the time/most of the time/some of the time/almost none of the time) --> binary (top 2 categories collapsed) | • Chi-square tests • Logistic regressions | • Lower prevalence of loneliness is found in Northern European countries and higher in the Mediterranean countries • Female sex is significantly positively associated with the likelihood of being lonely only in Spain, France and Greece • There is no significant association between age and likelihood of loneliness, except for Sweden, where it is positive • Education is linked to a lower likelihood of being lonely in France, Germany, Spain and Israel • Living with a spouse only is strongly linked to a lower likelihood of feeling lonely compared to living alone consistently across countries, while other living arrangements are linked to a lower likelihood of being lonely in Belgium, France, Greece, Italy, Netherlands and Spain • Poor subjective health is linked to a higher likelihood of being lonely in all countries except Denmark, Italy, Sweden and Switzerland |
| Tapia-Muñoz et al. (2022) | • US and 16 European countries  • 2013-2014  • Health and Retirement Study (HRS) (US), English Longitudinal Study of Aging (ELSA) (UK), and Survey of Health, Ageing and Retirement in Europe (SHARE) (15 countries)  • Cross-section  • N=75,891 (aged 50+, 56% female) | Risk factors:  • Demographic (age, sex)  • Socio-economic (education, work status)  • Marital status  • Health (self-reported, functional limitations, pain, depressive mood)  • Country-level (income inequality, gross domestic product per capita adjusted by power purchase parity)  Loneliness measure: R-UCLA 3-item scale --> continuous (3-9 score), binary (score 6 or more) | Hierarchical logistic regressions | • Divorced, widowed or single older adults are more likely to feel lonely, as well as those with physical health problems and especially and depressive mood  • The younger of older adults and those working are less likely to feel lonely  • Education is not significantly associated with loneliness  • Older adults living in more economically unequal countries are more likely to report loneliness and the relationship between country-level economic inequality and loneliness is independent of individual-level compositional factors and the GDP (which does not have a statistically significant relationship with loneliness) |
| ten Kate et al. (2020) | • Netherlands • 2016-2017 • Social Domain Index survey • Cross-section • N=7,920 (aged 40+) | Risk factors:  • Demographic (age, sex, ethnicity) • Socio-economic (employment, perceived financial situation, income) • Social (satisfaction with social relationships, participation in social activities - outside household, in clubs, social contacts - household composition, frequency of contact with family, friends, neighbours, relationship with a partner) • Health (perceived, chronic disease, difficulties with daily activities, use of formal care)  Loneliness measure: 6-item de Jong Gierveld scale --> 0-6 score for overall loneliness, 0-3 scores for emotional and social loneliness | • Bivariate analyses • Stepwise linear regressions | • Migrants have higher levels of both emotional and social loneliness than natives, but in the interaction model, the effect of migrant status becomes negative for all types of loneliness - there is an interaction between migrant status and satisfaction with social relationships (satisfaction protects against loneliness, but to a lesser extent for migrants) • Being a women is linked to higher emotional loneliness, but lower social loneliness • Being employed, having income difficulties (but not household income), bad perceived health, chronic diseases, contact with family and neighbours, bad relationship with a partner, social activities and social satisfaction all have significant positive effects on all types of loneliness • Difficulties with daily activities, living alone, not being socially active in clubs and lack of a good relationship with a partner have a positive effect only on emotional, not social loneliness • Age, living with a partner and contact with friends have a negative effect on social loneliness, but not on emotional loneliness • Formal care and having children in the household have no effect on loneliness |
| Tonković et al. (2021) | • 13 European countries  • 2019  • International Social Survey Program (ISSP)  • Cross-section  • N=14,588 (aged 25+, 53.4% female) | Risk factors:  • Demographic (age, sex)  • Socio-economic (education, ability to make ends meet, employment status)  • Social (living alone, personal network size and frequency of contact, social participation, sociability with friends, conflicts and poor quality in personal relationships, religious affiliation)  • Interpersonal trust  • Size of community  • European macro-region  Loneliness measure: UCLA 3-item scale --> score | OLS regressions | • Loneliness lowest in countries of Continental Europe compared to Nordic and Eastern European countries  • Men are less lonely than women only in Nordic countries, while participation in leisure, sports and culture is linked to less loneliness in Nordic and Eastern European countries  • Younger age is associated with more loneliness in all countries except those from the Continental bloc, being unemployed everywhere except the Central and Eastern European bloc, participation in political parties and voluntary organisations and being a Protestant (compared to Catholic) in Eastern European countries, and lack of trust in people only in the Continental bloc  • Education and community size are not linked to loneliness  • Worse financial situation, living alone and conflict in social relations correlate with loneliness across Europe (the last being the strongest predictor), while frequency of contact with one’s social network and feeling that people try to be fair are associated with less loneliness in all countries |
| van den Broek & Grundy (2017) | • Netherlands • 2014-2015 • Families of Poles in the Netherlands Survey • Cross-section • N=1,129 (aged 20-58, 59.8% female, Polish-born, with at least one Polish parent) | Risk factors:  • Age • Socio-economic (education, financial strain, employment) • Presence and location of partner (no, yes but not in Netherlands, yes and in Netherlands) • Presence and location of children (no, yes all abroad, yes all in Netherlands, yes both) • Migration-related (length of residence, Dutch language proficiency) • Health status • Religiosity  Loneliness measure: 6-item de Jong Gierveld scale --> 0-6 score | • Wald tests • F-tests • OLS regressions | • For women's loneliness, there is no effect of presence and location of a partner, while for men, only the presence of a partner in the country is linked to lower loneliness • Loneliness is lower only if children live both in the country and abroad  • Having worse self-rated health and being in financial strain are linked to higher loneliness • For men, the length of the stay in the Netherlands is linked with increasing loneliness • There is no effect of age, employment, education, religiosity and Dutch language proficiency on loneliness |
| van Tilburg (2021) | • Netherlands  • 2019-2020 • Longitudinal Aging Study Amsterdam • Longitudinal (2 waves) • N=404 (aged 74-96, 53% women) | Risk factors:  • Demographic (age, sex) • Socio-economic (education, income) • Social (partner status, personal network size, daily contact with someone, social participation, church attendance) • Health (number of chronic diseases, physical functioning) • Psychological (mastery, self-esteem) • Urbanity  Loneliness measure:  • 11-item de Jong Gierveld scale and 3 single items --> social and emotional loneliness scores (single items added to emotional loneliness score) • 7-item existential loneliness scale --> overall score | • Confirmatory factor analysis • Regression analysis | • In cross-sectional analysis before the COVID-19 pandemic, living with a partner, and higher self-esteem were linked to lower levels of all types of loneliness, mastery to lower emotional and social loneliness, daily contact with someone to lower social loneliness, bigger network size to less social and existential loneliness, and social participation to lower existential loneliness • Living with a partner, higher mastery and better physical functioning before the pandemic predict lower loneliness of all types during the pandemic, church attendance predicts an increase in emotional loneliness during the pandemic, and higher social participation and age predict a decrease in social loneliness • Number of chronic diseases, education, income, urbanity and sex are not related to loneliness before or during the pandemic |
| van Tilburg et al. (2021) | • Netherlands  • 2019-2020 • Longitudinal Internet Studies for the Social Sciences panel • Cross-section • N=1,679 (aged 65-102, 49% female, community-dwelling) | Risk factors:  • Demographic (age, sex) • Socio-economic (education, income) • Social (living with a spouse/partner, contact frequency - also relative to before pandemic) • Self-rated health and change • Urbanity of hometown  • COVID-19-related (personal impact of pandemic, excess mortality in municipality, following guidelines, change in trust in institutions, quarantine, worry, likelihood of getting infected, coping strategies)  Loneliness measure: 6-item de Jong Gierveld scale --> 0-3 social and emotional loneliness scores | • t-tests • Ordinal logistic regressions | • Contact frequency with (grand)children and age have a negative impact on social, but not emotional loneliness • Losing social contacts, work and activities, and being worried about the pandemic have a positive effect on emotional, but not social loneliness • Factors associated with both types of loneliness are being affected by being less outdoors (positive), being in need of support but not receiving it (positive), being a woman (positive for emotional, negative for social) and living with a partner (negative) • Relative contact frequency with children, and frequency of contact with others, being personally affected by illness or death, other COVID-19-related variables, coping strategies, health, urbanity, education and income are not related to loneliness |
| Victor & Yang (2012) | • UK • 2006 • European Social Survey • Cross-section • N=2,393 (aged 15-97, living in the community) | Risk factors:  • Demographic (age, sex) • Social (household size, marital (partner) status, frequency of social contact with family, friends and others, having someone to discuss intimate matters with, self-reported social engagement compared to others of the same age) • Perceived physical and mental health  Loneliness measure: Single item: "… how much of the time during the past week you felt lonely?" (none or almost none of the time/some of the time/most of the time/all or almost all of the time) --> binary (top category vs others) | • Descriptive analyses • Correlation analysis (Gamma statistic, chi-square tests) | • There is a U-shaped relationship between loneliness and age - loneliness levels are higher for those aged <25 and >55 (or >75 for men) • Loneliness is more likely to be reported by women, by those with feelings of depression (the strongest relationship), lacking an intimate figure and with poor self-rated health • Loneliness is less likely for those engaging more in social activities (also compared to others), lacking chronic diseases, being married, having tertiary education and with bigger household size • Only depression is relevant for loneliness across all age groups (and the effect is of the same magnitude) • Sex, marital status, household size, education, social contact relative to others and confiding relationship are significantly related to loneliness in all except young adults  • Physical health (both self-reported and presence of chronic illness) and frequency of social contacts are important for loneliness in all except the older group |
| Visser & El Fakiri (2016) | • Netherlands • 2012 • General health questionnaire of Public Health Services  • Cross-section • N=20,047 (aged 19+) | Risk factors:  • Demographic (age, sex) • Socio-economic (education, perceived financial situation, employment status) • Social (living with a partner, volunteer work) • Health (perceived, chronic disease, anxiety/depression) • Neighbourhood characteristics (deprivation, satisfaction, contact with neighbours, getting along with them, helping them) • Perceived discrimination  Loneliness measure: 11-item de Jong Gierveld scale --> scores for social and emotional loneliness | • Structural equation modelling • Regression analysis | • Loneliness is the highest among Turkish people, similar (and significantly lower) among Moroccan and Surinamese people, and the lowest among the Dutch, however, the mediation analysis found that the direct effect of ethnicity is negative, i.e., after taking into account all risk factors, the minorities experience less loneliness than the Dutch • For emotional loneliness, age, partnership, financial situation, living on welfare and getting along with neighbours have a differential impact on loneliness based on ethnicity  • For social loneliness, the effects of partnership, education and financial situation on loneliness depend on ethnicity  • Anxiety/depression, bad perceived health, not being satisfied with one's neighbourhood and less frequent contact with neighbours are universal predictors of loneliness • The largest indirect effects on loneliness for the minority ethnicity groups come from perceived health, anxiety/depression, perceived discrimination and having none/only primary education |
| Völker (2023) | • Netherlands • 2019-2020 • Longitudinal (2 waves) • N=1,342 (aged 18-35 and 65+, 55.7% female) | Risk factors:  • Demographic (age, sex) • Socio-economic (education, income, work situation) • Social (living situation, core discussion network, practical helpers network) • Self-rated health  Loneliness measure: Shortened version of de Jong Gierveld scale --> loneliness score | Mixed effects models | • Increase in loneliness linked to being male, living alone, having a low income, being unemployed, or suffering from worse health • Education is unrelated to changes in loneliness • Decline in both types of social networks is related to increases in loneliness, in particular the practical helpers network |
| von Soest et al. (2018) | • Norway • 2002-2007 • Norwegian Life Course, Aging, and Generation study • Longitudinal (2 waves) • N=5,555 (aged 40-79 at first wave, 51.4% female, non-institutionalised) | Risk factors:  • Demographic (age, sex) • Socio-economic (education level, income, unemployment, receiving a disability pension) • Social (frequency of contact with friends, partner status - cohabitation, divorce, widowhood, having children) • Having experienced a long sick leave • Big Five personality traits  Loneliness measure:  • Single item: "Do you feel lonely?" (never/seldom/sometimes/often) --> mean level and change • 3-items from de Jong Gierveld scale --> mean level and change | Structural equation modelling | • With the direct measure, women's loneliness increases quite steadily with age, while men's displays a U-shaped pattern - hence sex differences get stronger with age (but if other covariates are controlled for, sex differences disappear); with the indirect measure, there are no sex differences • Overall loneliness decreases with age and the decline is steeper in younger ages and men when direct measure is used; with indirect measure the decrease is present only at younger ages and there is an increase in older ages • Disability is linked to higher levels of loneliness using both measures, while education only if direct measure is used and unemployment only with indirect measure • Higher income is linked to a decrease in loneliness over time with both measures • Lack of spouse/partner, divorce and widowhood are positively linked to initial levels of loneliness (divorce only if indirect measure is used), while lack of spouse/partner and divorce are also linked to a steeper increase in loneliness if the direct measure is used • High contact with friends is linked to lower loneliness levels, and a steeper decline if loneliness is measured indirectly • Emotional stability and extroversion are both linked to lower levels of loneliness and steeper declines (extroversion only if using the direct measure of loneliness), while conscientiousness is linked to lower levels of loneliness only with the direct measure • Being on a sick leave, having children, agreeableness and openness are not related to initial level or change in loneliness over time |
| Warner & Kelley-Moore (2012) | • US • 2005-2006 • National Social Life, Health, and Aging Project • Cross-section • N=1,500 (aged 57-85, 44% female, married, community-dwelling) | • Demographic (age, sex, ethnicity) • Socio-economic (education, household income, working for pay) • Partner-related (number of previous marriages, cohabitation, marital quality - positive, negative) • Social (frequency of religious attendance, social network size) • Disability (number of functional limitations)  Loneliness measure: UCLA 3-item scale --> 0-6 score | Tobit regression models | • Functional limitations have a strong positive effect on loneliness robust to model specifications, as well as negative marital quality, while positive marital quality has a negative effect on loneliness • Positive marital quality, but not negative, moderates the effect of functional limitations of loneliness (similarly for men and women) - those with positive marital quality above average are protected from the effect of functional limitations, while the effect of functional limitations for those below average is worsened • Age and income have a negative effect on loneliness, Black ethnicity (compared to White) and having been married before have a positive effect and sex, Hispanic ethnicity (compared to White), education, social network size, working and religious attendance do not have any effect on loneliness |
| Wickens et al. (2021) | • Canada • 2020 • Cross-section • N=3,012 (aged 18+, 49.9% female) | Risk factors:  • Demographic (age, sex) • Socio-economic (education, income) • Social (marital status, living alone) • Current pandemic employment situation  Loneliness measure: Single item: "In the past 7 days, how often have you felt lonely?" (Rarely or none of the time (less than 1 day)/some or a little of the time (1–2 days)/occasionally or a moderate amount of the time (3–4 days)/most or all of the time (5–7 days)) --> top category vs others | • Chi-square tests • Multivariable logistic regressions | • Age and being married are linked to a lower likelihood of feeling lonely • Living alone and being unemployed because of the pandemic are linked to a higher likelihood of feeling lonely, while not working before the pandemic already or working from home are not linked to the likelihood of feeling lonely • Income and education are not associated with the likelihood of being lonely • Being a women is linked to a higher likelihood of feeling lonely only in the youngest (18-29 years) and oldest (60+) age categories |
| Wilson-Genderson et al. (2021) | • US • 2006-2020 • Ongoing Research on Aging in New Jersey: Bettering Opportunities for Wellbeing in Life • Longitudinal (4 waves) • N=2,458 (aged 50-74, 64% women) at baseline, N=3,076 at wave 5, N=3,137 at wave 6, N=2,458 at wave 7 | Risk factors:  • Demographic (age at baseline, sex, race) • Socio-economic (income at baseline, education) • Social (marital status at last wave, living alone at last wave, perceived social support at last 3 waves, social isolation at last wave) • Chronic illness score at last wave  Loneliness measure: UCLA 3-item scale --> 0-9 mean score | • Bivariate correlations • Multilevel mixed effects models | • Loneliness is lower and decreases over time for those living with others compared to those living alone (the decrease is driven by men living with others, while other groups experience an increase in loneliness, especially women living alone) • Younger age, male sex, lack of social support, social isolation and health conditions predict loneliness for any living arrangement, while higher education and lower income only for those living with others but having only small effects • Being married predicts less loneliness for those living with others (it is not measured for those living alone) |
| Yang et al. (2020) | • UK • 2015 • 6th survey of Millennium Cohort Study • Cross-section • N=11,872 (aged 14, 50.5% female) | Risk factors:  • Social (social support, having a best friend, frequency of arguing with each parent, frequency of being bullied by siblings, other adolescents and others online) • Psychological (negative feelings, self-harm, coping strategies for worry)  Loneliness measure: Single item: "I felt lonely" [over the past two weeks] (not true/sometimes true/true) --> categories or 1-3 score | • Cross-tabulations • Kendall's tau-b | • Girls report more loneliness than boys (almost twice as much) • Adolescents of white or mixed race are lonelier than others • All negative feelings are significantly positively correlated with loneliness, as well as self-harm in the past year, higher frequency of arguing with parents, of being bullied by others, especially peers and not having a close friend  • Social support is negatively associated with loneliness |
| Yang & Victor (2011) | • 25 European countries • 2004-2005 • European Social Survey • Cross-section • N=995 to 2,915 based on country (aged 15+) | Risk factors:  • Age • Nationality  Loneliness measure: Single item: "… how much of the past week you felt lonely" (none or almost none of the time/some of the time/most of the time/all or almost all of the time) --> categories or binary (top 2 categories collapsed) | • Descriptive analysis • Correlation analysis | • There is no universal pattern of the relationship between prevalence of loneliness and age across European nations • Countries can be grouped into 3 categories - (i) Eastern European countries, with loneliness rates increasing almost linearly with age (and also with the highest levels of loneliness), (ii) North and Western European countries, with loneliness rates quite stable throughout lifetime and increasing only after 70 years of age (also with the lowest levels of loneliness), and (iii) a mix of countries (mostly Southern European), with a gently U-shaped relationship between age and rates of loneliness |

**Table A4**

*Summary of Meta-Analyses*

| **Study** | **Data** | **Risk factors of interest & Loneliness measure** | **Method(s)** | **Findings** |
| --- | --- | --- | --- | --- |
| Buecker et al. (2020) | • 26 countries  • 1980-2018 • 113 studies in English or German • 1,697 effect sizes • N=93,668 individuals (Mage=12 to 100, 0-100% female) | Risk factors: Big Five personality traits (extraversion, agreeableness, conscientiousness, neuroticism, openness)  Loneliness measure: Single items and scales | Meta-analyses | • All five personality traits are related to loneliness - extroversion and neuroticism have a large effect (negative and positive, respectively), conscientiousness and agreeableness a medium negative effect, and openness a weak negative effect • When controlling for other personality traits, openness is not significantly related to loneliness anymore and the other relationships get a bit weaker • The negative relationship between extroversion and loneliness becomes weaker with age, while that between openness and loneliness becomes stronger with age • Relationships between both extroversion and neuroticism with loneliness are found to be weaker in more recent publications • Loneliness scale used moderates all relationships except that between openness and loneliness - associations are found to be stronger with the UCLA scale • Sex does not moderate the effects between personality traits and loneliness • Emotional loneliness has a weaker relationship with extroversion than social loneliness |
| Maes et al. (2019) | • 45 countries • 1978-2016 • 575 studies in English, French, German or Dutch • 751 effect sizes • N=399,798 individuals (Mage=5 to 90, 54.4% female) | Risk factor: Sex  Loneliness measure: Standardised questionnaires (scales) | Multilevel meta-analysis | • Most effect sizes for the relationship between sex and loneliness are close to zero • Overall, men are slightly lonelier than women, but when looking only at studies with larger sample sizes (at least 100 in each group), the effect is not significant • There is a number of moderators of the relationship between sex and loneliness - age (sex differences in loneliness are significant only in children, adolescents and younger adults), year of publication (sex differences in loneliness are smaller in more recent publications), sampling area (largest sex differences in loneliness are found in studies sampling from one city, compared to multiple cities/areas) • Loneliness type, relationship type, individualism of the country, socio-economic status, ethnic and clinical status do not moderate the relationship between sex and loneliness |
| Mahon et al. (2006) | • Multiple countries • 1980-2004 • 95 studies in English  • 9-33 hypotheses tested, depending on the risk factor • N=1,090 to 17,691, depending on the risk factor (aged 11-23) | Risk factors:  • Demographic (age, sex) • Social support • Maternal and paternal expressiveness • Psychological (depression, self-esteem, social anxiety, shyness, stress, self-disclosure)  Loneliness measure: Scales | Meta-analyses | • Large effect sizes for the relationship with loneliness are found for depression (positive), shyness (increasing with the study quality, positive), sex (usually boys lonelier than girls, but many studies find non-significant effects, so no conclusions should be drawn) • High medium effect size is found for self-esteem (increasing with increasing sample size, negative) • Medium effect size is found for social support (negative), social anxiety (positive), maternal and paternal expressiveness (negative) • Low effect size is found for stress (positive) and self-disclosure (negative) • Very low effect size is found for age (positive, but no conclusions can be drawn) |
| Mund et al. (2020) | • Multiple countries • Until 2018 • 75 longitudinal studies • 208 effect sizes • N=83,679 individuals (Mage=6+, 54.4% females) | Risk factor: Age  Loneliness measure: Single items and scales | Meta-analyses | • Cross-sectional age differences in loneliness show a slight U-shaped pattern, with loneliness decreasing through middle-age and then increasing in older age • Interindividual differences in loneliness are stable over 1 year and more volatile over 5 years and this stability in interpersonal differences in loneliness shows a reversed U-shaped pattern across life-span - it increases until old age and then sharply decreases • Mean levels of loneliness are quite stable across life-span, with a slight decrease in childhood and oldest old age - but if age is used as a continuous predictor there is no trend whatsoever, indicating that changes in loneliness are not due to age but individual experiences • Mean levels of loneliness across life-span are not moderated by cohort or continent, but are moderated by a percentage of females in the sample (more female-heavy samples document slightly larger decreases in loneliness over 5 years) and measurement type (increase in loneliness over time is found with indirect measures) |
| Pinquart & Sörensen (2001) | • Multiple countries • 1948-1999 • 149 studies in English, French or German, with mean age >=50  • 9-235 effect sizes, depending on the risk factor • N=3,814 to 93,934 individuals, depending on the risk factor | Risk factors:  • Demographic (age, sex) • Socio-economic (education, income, occupational status) • Social (size of social network, frequency of contact, close relationship with someone, emotional support) • Health (ADL, IADL, mobility, watching TV, other leisure activities) • Institutionalisation  Loneliness measure: Single items and scales | Meta-analyses | • Higher socio-economic status is linked to lower levels of loneliness - the effect is stronger for self-reported measures compared to the UCLA scale and stronger for income than education • There is a U-shaped relationship between age and loneliness - there is a decrease in loneliness in the youngest old, no effect of age in the middle-old category and increase in loneliness in the oldest old • Higher loneliness is associated to being a woman - no difference is found in studies using de Jong Gierveld scale and for unmarried samples • Low quality of social relationships is more strongly related to higher loneliness than low quantity of social relationships  • Low level of contact with neighbours and friends is more strongly linked to loneliness than contact with family (adult children as well) • Lower levels of competence and watching TV more are linked to more loneliness • More loneliness is found in old adults living in nursing homes compared to community-dwelling people, but there are only few studies on this |
| Schwartz-Mette et al. (2020) | • Multiple countries • Up to 2018 • 233 studies • 589 effect sizes • N=1,229 to 15,293, depending on the risk factor (school-age through adolescence) | Risk factors:  • Number of friends • Positive and negative friendship quality  Loneliness measure: Not specified | Meta-analyses | • Small to medium effect sizes are found for all three relationships between friendship indicators and loneliness cross-sectionally - number of friends and positive relationship quality have a negative relationship with loneliness, while negative relationship quality has a positive relationship with loneliness • These effects are confirmed (though with small to small-medium effect sizes) by longitudinal analyses (though only one study looks at negative relationship quality and loneliness longitudinally) • Relationships are also found to be significant in the opposite direction - loneliness impacts the number of friends and friendship quality longitudinally (though again, there is only one study on negative friendship quality and subsequent loneliness) |

**Table A5**

*Summary of Literature Reviews*

| **Study** | **Data** | **Risk factors of interest & Loneliness measure** | **Method(s)** | **Findings** |
| --- | --- | --- | --- | --- |
| Astell-Burt et al. (2022) | • Multiple countries  • 2000-2021  • 22 studies in English | Risk factors:  • Objective measures of green space (percentage of or distance to green space, residential greenness, member of allotment sites, viewing nature imagery, participation in activities with exposure to green space)  • Subjective measures of green space (perceived amount of, contact with, time spent and frequency visiting green space, access to and walking distance to green space, having outdoor area or green view from home or from the window, duration and frequency of viewing green space from window, time noticing nature, nature connectedness and relatedness, type of green space use)  Loneliness measures: UCLA or other loneliness scales, social isolation/disconnectedness | Systematic review | • The number of studies on the relationship between loneliness and green space is low and most have only fair quality  • More green space exposure or experience is associated with less loneliness, even though the relationship is often not statistically significant  • Very low number of longitudinal studies are available, but they confirm the negative relationship between green space and loneliness |
| Bower et al. (2023) | • Multiple countries  • 2002-2022  • 57 studies in English  (17 qualitative, 36 quantitative, 4 mixed-methods) | Risk factors:  • Built environment (neighbourhood characteristics, urbanicity or city design, neighbourhood social context, housing design/conditions, qualitative housing aspects, natural spaces)  Loneliness measures: UCLA scale, de Jong Gierveld scale, single item measures | Systematic review  & narrative synthesis | • In terms of structured environment, the macro-level urban characteristics such as neighbourhood density, city size, and urbanicity have mixed effects on loneliness, while public spaces and amenities and greater exposure to natural spaces seem to be linked to decreased loneliness  • In terms of lived environment, a sense of neighbourhood and positive neighbourhood perception are linked to reduced loneliness whereas perceived threats from crime and antisocial behaviour are associated with increased loneliness  • The relationship between built environment characteristics and loneliness is often mediated by the socio-economic characteristics of the respondents and/or the cultural context |
| Cohen-Mansfield et al. (2016) | • Multiple countries • 2000-2012 • 38 studies in English, with N>= 100 and participants of Mage >=50 | Risk factors:  • Demographic factors (age, sex) • Socio-economic (income, education) • Social factors (living alone, marital status, quality and quantity of relationships, social opportunities, having children, emotional/social support, caring responsibilities) • Health (self-reported, chronic illness, doctor visits, functional limitations, sensory and mobility problems, unhealthy behaviours) • Psychological (mental health, life satisfaction, depression, self-esteem/efficacy, cognitive deficit, negative life events) • Residency  Loneliness measure: Not specified | Review | • There are mixed results on the relationship between age and loneliness - both positive and negative, possibly U-shaped • Women are quite consistently found to be lonelier than men, but sometimes when other factors are controlled for the relationship between sex and loneliness vanishes  • Unmarried status is consistently linked to higher loneliness, especially when it comes to widowhood, and so is living alone, lower education, lower income, poor self-reported health, functional limitations, and depression • Few studies find evidence of higher loneliness in old adults living in nursing/residential care homes and rural areas • Some studies find evidence that lower quality and quantity of social relationships, less emotional and social support, and having no or fewer children are linked to higher loneliness, and similarly low self-esteem/efficacy, psychological distress or low life satisfaction |
| Dahlberg et al. (2021) | • 11 OECD countries • 1999-2018 • 34 longitudinal studies in English, with participants of Mage >=60 | Risk factors:  • Demographic (age, sex, ethnicity) • Socio-economic (financial situation, education, social status, employment status) • Social (quantity and quality of social networks, marital/partner status, living arrangements, social support, social activity) • Health (self-reported, functional limitations, cognitive functioning) • Psychological (e.g. depression)  Loneliness measure: Single items and scales | Systematic review | • Risk factors consistently associated with higher loneliness across studies are not having a partner/not being married, partner loss, limited social network and social activity, poor self-perceived health, depression/depressive mood and increase in these • Evidence for the effect of social support on loneliness is inconsistent and the same holds for functional limitations  • There are indications that low income (or poor financial conditions) is linked to higher loneliness, while education and loneliness are mostly unrelated  • There are positive associations between age and female sex and loneliness in bivariate analysis, but they mostly disappear in multivariate analyses |
| Lyu & Forsyth (2021) | • Multiple countries • Up to 2020 • 36 studies in English, with participants aged 50+ | Risk factors:  • Neighbourhood characteristics (local resources and daily destinations, walkability, green space, overall neighbourhood environment) • Urban context (degree of urbanisation, urban/rural area) • Housing (institutionalised living, age-specific housing, type of dwelling)  • Transportation  Loneliness measure: Not specified | Scoping review | • Loneliness is linked to lower quality of built environment in general, but also specifically to worse perceived/self-reported neighbourhood resources (but not in all studies), perceived worse walkability (but not when this is measured objectively), less access to or time in green spaces (though there is not much evidence available) and difficult access to transportation • There is no effect of urban vs rural environment or degree of urbanisation on loneliness when other environmental factors are controlled for • There are mixed findings for the type of dwelling and housing |
| Morrish & Medina-Lara (2021) | • Higher income Western countries in Europe, North America and Oceania • Up to 2020 • 37 studies in English, with participants aged 16-65 | Risk factor: Employment status  Loneliness measure: Single items and scales | Systematic review | • Employment is potentially a protective factor against loneliness, while unemployment is associated with higher loneliness • Causal studies suggest that this relationship is potentially bidirectional, but more evidence is needed to prove this • Different studies find different age groups to have the strongest relationship between loneliness and employment |
